# Displacement-pressure biparametrically regulated softness sensory system for intraocular pressure monitoring

**DOI:** 10.1093/nsr/nwae050

**Published:** 2024-02-06

**Authors:** Yu Cheng, Yifei Zhan, Fangyi Guan, Junli Shi, Jingxiao Wang, Yi Sun, Muhammad Zubair, Cunjiang Yu, Chuan Fei Guo

**Affiliations:** Department of Materials Science and Engineering, Southern University of Science and Technology, Shenzhen 518055, China; Department of Materials Science and Engineering, Southern University of Science and Technology, Shenzhen 518055, China; Department of Materials Science and Engineering, Southern University of Science and Technology, Shenzhen 518055, China; Department of Materials Science and Engineering, Southern University of Science and Technology, Shenzhen 518055, China; Department of Materials Science and Engineering, Southern University of Science and Technology, Shenzhen 518055, China; Department of Materials Science and Engineering, Southern University of Science and Technology, Shenzhen 518055, China; Department of Engineering Science and Mechanics, Pennsylvania State University, State College 16802, USA; Department of Engineering Science and Mechanics, Pennsylvania State University, State College 16802, USA; Department of Biomedical Engineering, Pennsylvania State University, State College 16802, USA; Department of Materials Science and Engineering, Materials Research Institute, Pennsylvania State University, State College 16802, USA; Department of Materials Science and Engineering, Southern University of Science and Technology, Shenzhen 518055, China

**Keywords:** intraocular pressure, tonometer, deep learning, softness, iontronic sensor

## Abstract

High intraocular pressure (IOP) is one of the high-risk pathogenic factors of glaucoma. Existing methods of IOP measurement are based on the direct interaction with the cornea. Commercial ophthalmic tonometers based on snapshot measurements are expensive, bulky, and their operation requires trained personnel. Theranostic contact lenses are easy to use, but they may block vision and cause infection. Here, we report a sensory system for IOP assessment that uses a soft indentor with two asymmetrically deployed iontronic flexible pressure sensors to interact with the eyelid-eyeball in an eye-closed situation. Inspired by human fingertip assessment of softness, the sensory system extracts displacement-pressure information for soft evaluation, achieving high accuracy IOP monitoring (>96%). We further design and custom-make a portable and wearable ophthalmic tonometer based on the sensory system and demonstrate its high efficacy in IOP screening. This sensory system paves a way towards cost-effective, robust, and reliable IOP monitoring.

## Teaser

This work mimics the fingertips for softness sensing and designs a biomimetic softness sensory system for intraocular pressure monitoring.

## INTRODUCTION

High intraocular pressure (IOP) has been found to be associated with the abnormal circulation of aqueous humor and the monitoring of IOP has been an effective strategy to prevent eye-related diseases, including the acute angle-closure glaucoma [1], the second leading cause of irreversible loss of vision worldwide [2]. Currently, reducing IOP, the only known quantifiable hazard factor, is an effective method to prevent optic nerve damage and vision loss [3,4]. Therefore, daily and point-of-care monitoring of IOP is crucial to eye health, especially for glaucoma screening in high-risk groups [5].

The evaluation of IOP first originated from palpation to feel the hardness of the eyeball, credited to William Mackenzie [6] who discovered eyeball hardness as a feature of glaucoma in 1830. Considering the subjectiveness of palpation, objective approaches for snapshot-based IOP detection have been explored nowadays, among which the gold-standard medical measurement in hospitals is based on the Goldmann applanation tonometry that uses an indenter to directly interact with the cornea [7]. Despite the high accuracy, this technique is unavailable for daily use because of its reliance on a bulky benchtop device as well as professional clinicians [8]. In contrast to in-office assessments, portable home tonometers that provide convenient IOP monitoring are becoming popular. However, these devices often suffer from large errors (>5 mmHg) and discomfort since they still utilize an indentor to directly contact with the cornea [9]. Wearable soft contact lenses are another alternative that enables wireless and real-time monitoring of IOP with miniaturized integrations [10–13]. The lenses, however, may partly hinder vision, and like other direct cornea-interaction techniques, can introduce a risk of infection or abrasion to the corneas, not even mentioning the complexity and dedicated sensing and electronic modules for untethered readout and that a significant proportion of the population cannot wear contact lenses [10,11].

Here, we develop a palpation-type sensory system, which uses two asymmetrically deployed iontronic flexible pressure sensors with a high sensitivity of 736.1 kPa^–1^ integrated into a soft hemispheric indentor to interact with the eyelid of a closed eye for real-time and high-accuracy IOP evaluation. Inspired by softness assessment with a human fingertip, this sensory system detects the softness (in this work we use the term softness because the system can discriminate deformable materials only) of the eyeball by extracting force and displacement information measured by the two sensors during the interaction between the soft indentor and the eyelid. The high-performance sensors enable a quick and easy capture of the feature information to construct a machine-learning model for softness evaluation. This system is operated without any damage or contamination to the cornea, while the testing accuracy and testing consistency are far higher than that of commercial tonometers, and it shows high robustness and efficiency under different temperatures, humidities, and loading conditions. A custom-made, portable, wearable ophthalmic tonometer based on the sensory system has been further developed and has exhibited high efficacy in IOP monitoring and screening.

## RESULTS AND DISCUSSION

### Design and working principle of the tonometer

**Figure 1.**
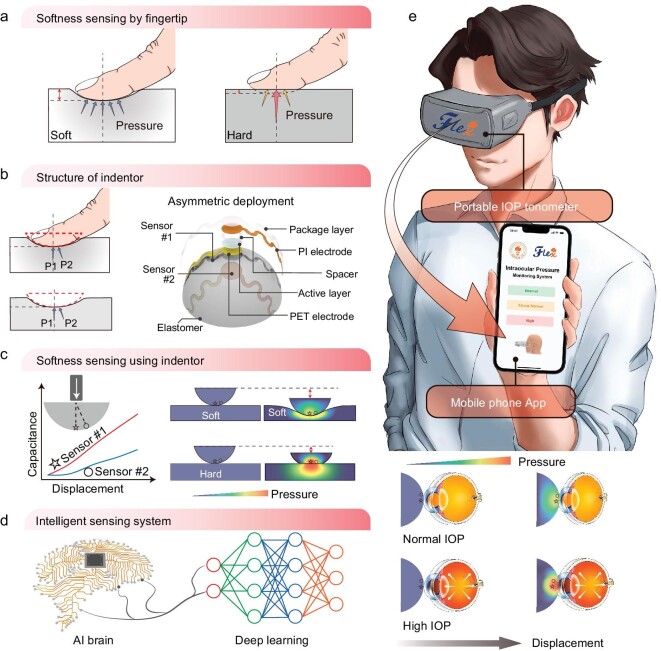
Design and principle of the portable IOP tonometer. (a) Mechanism for the feel of softness by a human fingertip. (b) Structure of the indentor. Two sensors are integrated on the sphere surface. (c) Muti-segment displacement control of the indentor. (d) Deep learning enabled intelligent sensing system. (e) Schematic of the portable IOP tonometer.

Our sensory system for softness test is inspired by the human sensory system, which often feels softness by touching an object with a fingertip. The nerves and the brain are also involved in signal transmission and information processing, respectively. Mechanoreceptors of a human fingertip are distributed on a curved surface. The feeling of softness often involves a group of mechanosensors that ‘detect’ both pressure and displacement upon pressing. The central part of the touch often has a higher pressure than the marginal part. A relatively homogeneous distribution of pressure in addition to a large displacement give a feel of high softness of the object, while a larger central-to-marginal pressure difference and a smaller displacement give a feel of ‘hard’ (Fig. 1a).

Here, we design a finger-like indentor for softness sensing. Unlike general approaches that pay much effort to detect numerous physical parameters while the detecting range is often limited (Fig. S1 and Note S1), in the indentor, we use two sensors with asymmetric deployment—one (sensor #1) placed on the pole of an elastomeric hemisphere, and the other (sensor #2) placed at an angle of 25° to the principal axis (Fig. 1b), to detect the contact pressures and deflection from the center. Only two sensors are needed to collect the pressure information for softness sensing because of the symmetry of the hemisphere and the uniformity of the contact. The detailed fabrication of the sensors is described in Fig. S2. When the indentor is pressed against and makes contact with a soft object, featured sensing information—contact force as a function of displacement can be captured (Fig. 1c and Fig. S3), while the two sensors show pressure to displacement response. This system can recognize softness based on a pressure-displacement coupled algorithm (Fig. 1d). We further design a wearable IOP tonometer based on the sensory system, for which the signal is transmitted to a cellphone and the result of IOP evaluation is reported in a mobile phone app (Fig. 1e).

### Properties of the pressure sensor

**Figure 2.**
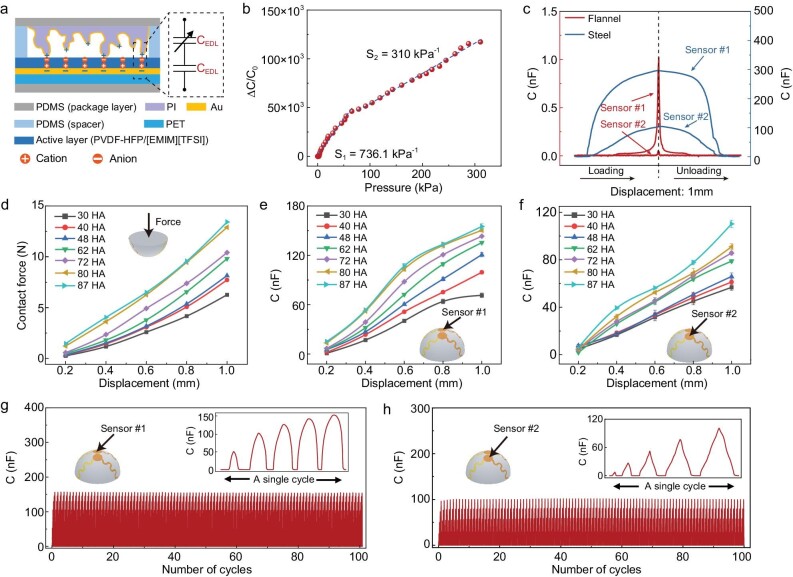
Sensing properties of the sensors. (a) Schematics showing the cross section of the iontronic sensor and its working principle. (b) Response of the sensing unit to pressure in the range of 0 to ∼300 kPa. (c) Distinct capacitive signals of the two sensors when touching steel (modulus: ∼200 GPa) and velvet (modulus: <1 kPa) at a same displacement of ∼0.1 mm. (d) Contact force as a function of displacements (at 0.2, 0.4, 0.6, 0.8, and 1.0 mm) by touching seven materials with different Shore hardness values. (e and f) Capacitance as a function of displacement for sensor #1 and #2 by touching the seven different samples. (g and h) Signals of the two sensors when they are in contact with a piece of block (87 HA) in a multi-segment displacement mode over 100 cycles.

The sensor consists of an ionic active layer (poly(vinylidene fluoride-co-hexafluoropropylene) (PVDF-HFP)-1-ethyl-3-methylimidazolium bis(trifluoromethylsulfonyl)imide ([EMIM][TFSI])) (Fig. S4) sandwiched in between a flat polyethylene terephthalate (PET)-gold (Au) electrode and a microstructured PI (polyimide)-Au electrode (Fig. 2a), governed by the iontronic sensing mechanism [14]. The PI membrane has a microstructure (called graded intrafillable architecture [15]) (Fig. S5) that leads to both high sensitivity (736.1 kPa^–1^ in 0–60 kPa, and 310.1 kPa^–1^ in 60–300 kPa, Fig. 2b) and a rapid response-relaxation speed (response time: 5.4 ms, recovery time: 6.4 ms, Fig. S6). Our iontronic sensor exhibits a high sensitivity and a wide working range; such high sensing properties are crucial to the performance of the sensory system.

When the hemisphere makes contact with a target object at a given displacement, each sensor records a pressure that is dependent on the softness of the object. For example, the signal of touching a piece of hard steel (Young's modulus *E* ∼200 GPa) varies substantially from that of touching soft flannel (*E* <1 kPa) in terms of the shape of the signal and the pressure-difference between the two sensors (Fig. 2c). Furthermore, the contact force increases with displacement when the indentor presses on soft materials with different Shore hardness values of 30, 40, 48, 62, 72, 80, and 87 HA (Fig. 2d and Fig. S7). We further record the capacitance-displacement curves of the two sensors. Such data provide rich information to distinguish objects with different Shore softness values for us to further establish an efficient deep learning model (Fig. 2e and f). In addition, the iontronic pressure sensors were verified to have high working stability, either in cyclic loading-unloading with a fixed peak pressure (over 5000 cycles, Fig. S8), or with each cycle a set of different displacements (Fig. 2g and h). The high stability might be derived from the high compression endurance of the PDMS hemisphere (Fig. S9) that guarantees data reliability.

### Deep learning enabled softness classification

**Figure 3.**
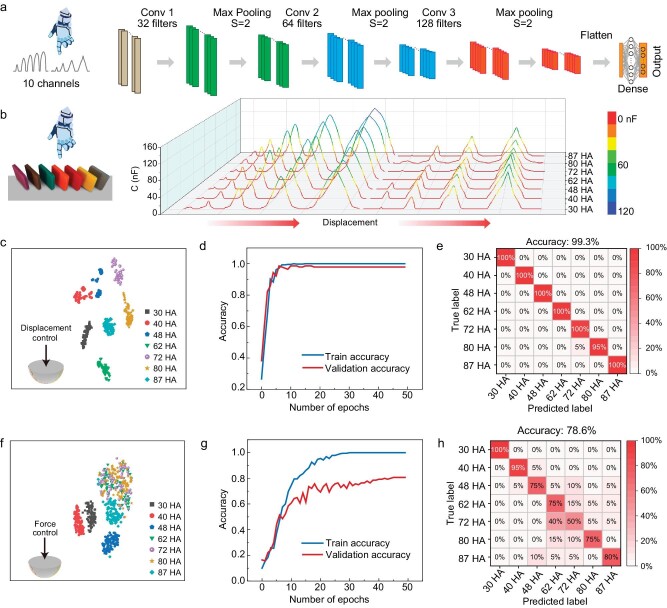
Construction, accuracy, and efficiency of the deep learning model for the sensory system. (a) Structure of a one-dimensional convolution neural network model used in this work. (b) A single dataset of the softness sensor touching objects with different softness values. Comparison of the distinguishability, efficiency, and accuracy of deep learning models using (c–e) displacement-pressure model and (f–h) only force-controlled model. Panels (c) and (f) show one-dimensional points after dimensionality reduction through t-SNE. Panels (d) and (g) show the accuracy of training and validation with different numbers of epochs, and panels (e) and (h) show confusion matrix of softness recognition.

We construct a neural network based on deep learning for softness classification. It has been verified that high-quality small data in combination with effective algorithms can serve as a powerful tool to construct a high-performance deep learning system [16]. A one-dimensional convolution neural network (1DCNN) with multiple channels was selected to construct a deep learning model by extracting and enlarging the features of the datasets (Fig. 3a), updated through backpropagation until the training loss is minimized (Fig. S10). Note that 1DCNN is commonly used in the field of intelligent sensing and has proven to be effective in deriving features from time-series data [17–21]. Detailed parameters used for this network are given in Table S1. The feature dataset is collected using the hemispheric indentor to press seven samples with different Shore hardness values (30, 40, 48, 62, 72, 80, and 87 HA) at five characteristic displacements (0.2, 0.4, 0.6, 0.8, and 1.0 mm). This method is defined as ‘multi-segment displacement control’, for which characteristic data are collected at stepwise displacements. For each sample, 100 sets of data were collected with each set containing 10 characteristic peaks from the two sensors (five for each sensor). The peaks were extracted from time series, resampled and converted into 10 separate channels. Consequently, within one single data sample, data points that correspond to the contact approximately align across channels. There are a total of 700 sets of data, containing 7000 characteristic peaks (one peak per channel). Sixty percent of the data is used for training, 20% for validation, and 20% for testing.

The softness of the objects can be well evaluated using the featured dataset under a multi-segment displacement control (Fig. 3b). T-distributed stochastic neighbor embedding (t-SNE) was utilized to visualize the data. The t-SNE method can reduce the dimensionality of extracted features and display the results in a 2D space, as shown in Fig. 3c. It shows that the normalized data of multi-segment displacement control from different samples can be well distinguished (Fig. S11a and Note S2). Therefore, sample points of the same object are clustered closely together, indicating that the hemispheric indentor is capable of accurately sensing the softness of objects through displacement control. Such a small but high-quality dataset contributes to the high training efficiency of the model—a validation accuracy of 99.3% is achieved within only 18 epochs (Fig. 3d and e). The validation accuracy and training accuracy are similar, which means that the training model is adaptive to unknown data. Therefore, our model has a high generalization ability.

The results of force-control mode (using a single parameter of force) reported in the literature were compared with that of our multi-segment displacement control [22,23]. With the absence of displacement parameters, data points are poorly clustered (Fig. 3f) because all the samples with different hardness values give similar outputs (Fig. S11b and c) and undistinguishably normalized data (Fig. S11d). Without displacement control, validation accuracy is much lower than the training accuracy (Fig. 3g), indicating low generalization ability. As a result, a much lower testing accuracy (78.6%, Fig. 3h) is achieved. We thus conclude that the introduction of displacement information substantially increases the classification accuracy.

Note that the two-sensor deployment and the multi-segment displacement strategy both help achieve a high accuracy in evaluating softness. The classification accuracy drops to 95.7% when using only sensor #1, and to 92.9% when using only sensor #2 (Fig. S12a and b). Likewise, the number of displacements used in the indentor-sample interaction also affects the accuracy: the model gives a 97.1% accuracy for the case of four displacements, and further decreases to 96.4% when only a single displacement is used (Fig. S12c and d).

Our sensory system, which consists of the indentor (with both force and displacement control), data collection, and the deep learning model, can be used for high-accuracy recognition of soft objects. We select 20 objects from soft elastomers in laboratories to leathers, foods, foams, and to many other commonly seen objects for our test (Fig. S13a and b). The training of the model is completed in only ∼12 epochs (Fig. S14a), and the clustering of t-SNE data points shows well-defined borders that are separated from each other (Fig. S14b). Accordingly, the classification accuracy reaches 99.25% (Fig. S14c). Such a model trained in only a few epochs while exhibiting a high recognition accuracy signifies a low computational cost as well as a high working efficiency, which is one to two orders of magnitude higher than that in previously reported sensory systems based on deep learning [17–21,24–27] (Fig. S15 and Table S2).

The high performance of the sensory system is contingent upon several factors: highly sensitive sensors that provide precise data, a specially designed data acquisition method that yields multi-feature data, and an elaborately designed deep learning model. While sensory systems based on flexible sensors and machine learning have already been reported [28,29], existing work often uses sensors to measure simple contact force (or pressure) [24,30], for which the data often fail to reflect the spatial characteristics of the object (caused by deformation), leading to a suboptimal training efficiency.

### Reliability and robustness of the sensory system

**Figure 4.**
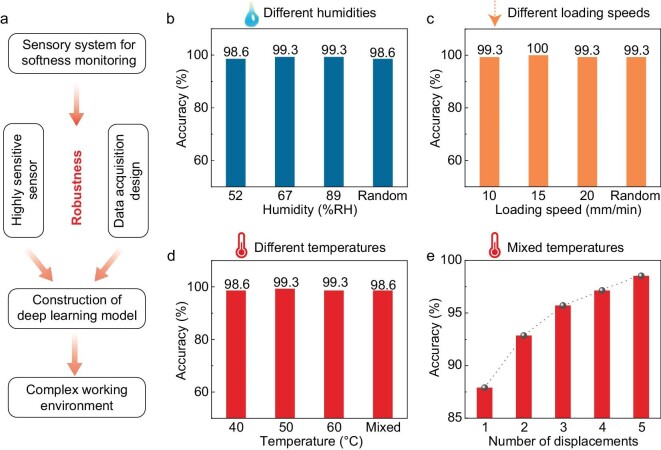
Reliability and robustness of the sensory system. (a) The sensory system for softness evaluation and its application under complex working conditions. Classification accuracy of the sensory system under different humidity conditions (b), loading speeds (c) and temperatures (d). (e) Effect of the number of displacements used on accuracy.

Our sensory system exhibits high robustness under complex working conditions, including varied humidities, temperatures, and loading speeds of the indentor (Fig. 4a). The indentor is first placed in a confined space for which the working humidity can be tuned. We show that there is no substantial change in the shape and magnitude of the signal for both sensors at relative humidity (RH) values of 52%, 67% and 89% RH (Fig. S16a–c). Correspondingly, the t-SNE data points remain well clustered and distinguishable (Fig. S16d), and the model gives a classification accuracy of at least 98.6% under different humidity conditions (Fig. 4b and Fig. S17a–c), and 98.6% under random humidity (Fig. S17d). The humidity-insensitive behavior stems from the hydrophobic nature of the ionic material used in this work (Fig. S18).

The classification accuracy is also insensitive to the loading rate of the indentor. The amplitude of the signal does not change with the loading rate, when the rate changes randomly in the range from 2.5 to 20 mm min^–1^ to interact with a sample of 30 HA and at a displacement of 1 mm (Fig. S19a–c). Because of the high-quality data sets collected in a displacement-control manner (Fig. S19d), an equally high classification accuracy of higher than 99% (Fig. 4c and Fig. S20a–d) is maintained. The result is related to the high response-relaxation speed of the sensors.

Although both the sensing properties of the sensor and the softness of materials may change with temperature, the sensory system remains effective in the identification of objects as temperature changes from room temperature to 60°C. Iontronic sensors often exhibit higher signal amplitude at higher temperatures (Fig. S21a) because of the increasing ion mobility [31], and this change can compensate for the change in signal in a temperature-changing process. For example, the signal corresponding to the sample of 30 HA at 60°C is similar to that of the sample of 40 HA at 40°C (Fig. S21b). The experimental results indicate that changes in temperature do not affect the ratio between the peak values measured at different displacements (Fig. S21c). The multi-segment displacement design ensures that the deep learning model not only considers the absolute values of each peak, but also the relationship between the peaks. Since one peak is converted to one channel, the design enables cross-calibration between channels. We thus trained a neural network based on the mixed data collected under varying temperatures (30, 40, and 50°C). The t-SNE plot reveals well-clustered data points (Fig. S21d), and the model classification accuracy is higher than 98% under different temperatures (Fig. 4d and Fig. S22a–d). We repeated the training process by using fewer displacements to further investigate the effect of the cross-validation, and the results show that the classification accuracy decreases with the decreasing number of displacements (Fig. 4e), verifying the necessity of temperature calibration through multi-segment displacement control.

### Portable IOP tonometer

**Figure 5.**
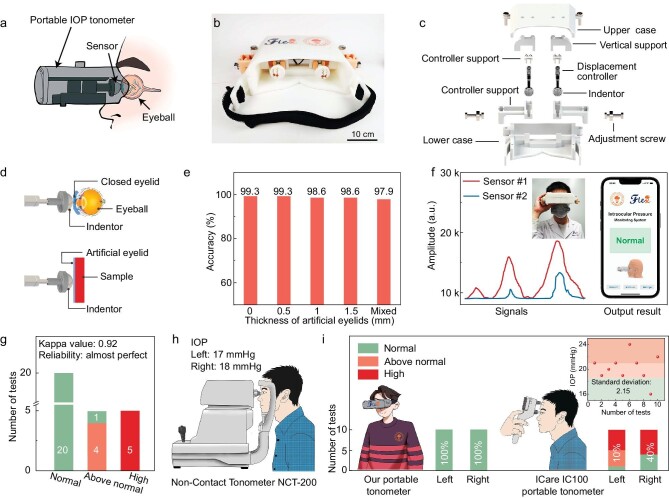
Structure of the portable tonometer and its application in IOP assessment. (a) Schematic diagram of the IOP assessment using our portable tonometer. (b) Photo of the portable IOP tonometer. (c) Components of the portable IOP tonometer. (d) Schematic of IOP evaluation by touching an eyelid using the indentor. A soft bilayer is used to simulate the eyelid. (e) Accuracy of softness classification when using an artificial eyelid of different thicknesses of 0.5, 1.0, and 1.5 mm. The case of no artificial eyelid is also involved. (f) An example showing the measured signals from the two sensors of an indentor and the final assessment using our system. (g) Deep learning results of IOP assessment. (h) Results of the jet measurement-based IOP tonometer (Non-Contact Tonometer NCT-200) from a subject. (i) Comparison of results between our portable IOP tonometer and a portable IOP tonometer (ICare IC100).

We have designed and custom-made a portable and wearable IOP tonometer (Fig. 5a and b) based on our sensory system. The tonometer has binocular indentors with displacement controllers (Fig. S23a and b) that are integrated into a 3D-printed headset shell, and two adjustment screws that help align with the eyeballs (Fig. 5c). Our portable tonometer costs only about 200 USD, which is more cost-effective compared with that of commercial portable tonometers (e.g. ICARE IC-100, which costs about 2000 USD). We have also designed a program to control the tonometer (Fig. S24). The signals are collected and processed using a circuit board, of which the details can be seen in the Methods section. Data are used to train a deep learning model serving as an ‘AI brain’. In real-time testing, data are sent to the ‘AI brain’ to make a classification and results are returned to the user via a mobile app (Fig. S25). In the tonometer, each indentor uses two sensors for pressure detection at three displacements of 1, 2, and 3 mm, using a micro linear meter that has a displacement accuracy of 0.2 mm.

Our sensory system can detect the softness of a material even if it is covered by another thin layer of a softer material—which is similar to the case when an eyeball is covered by an eyelid, allowing our system to conduct palpation on closed eyes for IOP monitoring (Fig. 5d). We used a layer of Ecoflex 00–30 (Young's modulus: ∼60 kPa) as the top soft layer that analogues an eyelid and placed the soft layer on samples of different hardness values. Here, we use a stable and softer elastomer (Young's modulus: 70 kPa, Fig. S26a and b) for the hemispheric indentor to protect the eyeballs (Young's modulus: >70 kPa) [32,33] and also to reduce the discomfort to the users. The sensing components exhibit an effective modulus of 340 kPa, and the sensor can survive at a radius of curvature down to 1.1 mm (Fig. S27a–b). The magnitude of force is within 0.6 N (30 kPa) during the IOP testing, and the force that causes eye pain is about 1 N [34]. Our IOP tonometer will not injure the human eyes. Our experiment shows that the signal magnitude decreases slightly with increasing thickness of the artificial eyelid at a small displacement, and this phenomenon becomes negligible as the displacement increases because the deformation is determined by the harder materials (Fig. S28a and b). The final classification accuracies under different thicknesses of the artificial eyelid maintained >97% (Fig. 5e and Fig. S29a–d). Similar results were obtained when the modulus of the artificial eyelid changes (Fig. S30a and b, Fig. S31a–d). The result indicates that the existence of eyelids will not have a substantial impact on the assessment of softness under large indenting displacements. In addition, our simulation shows that the indentor can also collect characteristic signals of curved objects to meet the needs of data acquisition on eyeballs (Fig. S32).

Users have their eyes closed during measurement, and get rapid feedback with the IOP assessment displayed in a mobile app (Fig. 5f, Fig. S33 and Movie S1–S3). There are three possible results for the assessment: normal (corresponding to 10–18.5–mmHg), above normal (18.5–21 mmHg), and high pressure (>21 mmHg). Three hundred data sets of 50 eyeballs (from 8 females and 17 males, with ages ranging from 20 to 60, Table S3) were used in the deep learning model for IOP assessment. The tested IOP values were in the range of 10 to 29 mmHg. Because there is often a low proportion of people suffering from high IOP, the presence of the minority classes (‘above normal’ and ‘high’) in the dataset can significantly affect the performance of the deep learning model, resulting in data imbalance. The entire dataset was split into training (70%), validation (15%), and test sets (15%) while retaining the original data distribution. An oversampling algorithm, synthetic minority oversampling technique (SMOTE), was applied to restore the balance of the training set. SMOTE creates synthetic minority class samples by interpolating new samples between existing ones in a feature space [35]. This algorithm balances the training set and prevents the deep learning model from being biased towards the majority class (normal IOP). Detailed parameters used for this model are shown in Table S4.

The kappa coefficient is a statistical measure used to quantify inter-rater reliability or agreement in classification tasks. By accounting for the likelihood of random agreement and penalizing biases towards large categories, it is particularly useful in contexts where imbalances may exist within the dataset (Note S3) [36]. A value of kappa coefficient close to 1 is indicative of high agreement. In our case, the determined kappa coefficient is 0.92 (Fig. 5g), which not only signifies that our model is highly reliable but also coincides with the model's impressive overall accuracy of 96.7%.

We have further verified the practicability and reliability of our portable tonometer in random tests and repeated experiments. Ten subjects were randomly selected for the validation of our system. Measured using a jet measurement-based commercial tonometer (Non-Contact Tonometer NCT-200), six out of the ten subjects fall in the normal region, while two subjects are in the ‘above average’ region, and the remaining two in the high IOP region. The IOP results assessed using our tonometer match well with that measured using the Non-Contact Tonometer NCT-200 (Fig. S34a and b). A volunteer subject was selected to verify the repeatability and accuracy of our portable IOP tonometer. The IOP values of the left and right eyes are determined to be 17 and 18 mmHg, respectively, using the NCT-200 tonometer in hospital (Fig. 5h). The values indicate both eyes are in a normal condition. We then used our tonometer to measure each eye for 10 times, and the results given were all normal, exhibiting a consistency of 100% compared with the results of the NCT-200 tonometer. Further, we used a widely used commercial portable IOP tonometer based on rebound measurement (ICare IC100) to detect the IOP values, while this facility gives significantly dispersed results with high standard deviations of 2.15 and 3.07 for the left and right eyes (inset in Fig. 5i and Fig. S35), and corresponding accuracies are 10% and 40% (Fig. 5i), respectively. The results indicate that our portable IOP tonometer exhibits far higher repeatability and consistency compared with commercial wearable devices.

## CONCLUSION

In this work, we demonstrate a palpation-type sensory system, which is inspired by the palpation of fingers, for IOP assessment based on asymmetric, two-point displacement-pressure control and deep learning. Our simple yet effective design can extract both displacement and pressure information, allowing for the capture of subtle changes in softness of materials. A small-dataset solution was used to deal with the softness classification problem in complicated conditions (with varied temperature, humidity, displacement, or an additional soft layer), and it shows high accuracy and high efficiency (over 99% accuracy within only 18 epochs) due to the capability of the system to capture rich feature information. Our study shows that improving the characteristic quality of sensing signals is a desired way to enhance the training efficiency and classification accuracy instead of making a larger and more complex machine learning model. Benchmarking our custom-made wearable tonometer, the results show high efficacy, reliability, and repeatability, compared with existing commercial wearable devices.

## MATERIALS AND METHODS

Detailed materials and methods are available in the Supplementary data.

## Supplementary Material

nwae050_Supplemental_Files
