# Supplementary material for: Displacement-pressure biparametrically regulated softness sensory system for intraocular pressure monitoring
Source: Natl Sci Rev. 2024 Feb 6;11(6):nwae050. doi: 10.1093/nsr/nwae050 (PMC11067962; doi:10.1093/nsr/nwae050)
Supplement: nwae050_Supplemental_Files [file nwae050_supplemental_files.zip › Supporting Information 0116.docx]

**Displacement-pressure biparametrically regulated softness sensory system for intraocular pressure monitoring**

Yu Cheng^1,†^, Yifei Zhan^1,†^, Fangyi Guan^1^, Junli Shi^1^, Jingxiao Wang^1^, Yi Sun^1^, Muhammad Zubair^2^, Cunjiang Yu^2,3,4,^*, Chuan Fei Guo^1,^*

^1^ Department of Materials Science and Engineering, Southern University of Science and Technology, Shenzhen, Guangdong 518055, P. R. China.

^2^ Department of Engineering Science and Mechanics, Pennsylvania State University, University Park, PA 16802, USA.

^3^ Department of Biomedical Engineering, Pennsylvania State University, University Park, PA 16802, USA.

^4^ Department of Materials Science and Engineering, Materials Research Institute, Pennsylvania State University, University Park, PA 16802, USA.

^†^ These authors contributed equally to this work.

* To whom correspondence should be addressed. Email: [cmy5358@psu.edu](mailto:cmy5358@psu.edu) (C.Y.) [guocf@sustech.edu.cn](mailto:guocf@sustech.edu.cn) (C.F.G.)

**Materials and Methods**

**Fabrication of the softness sensor**

A PDMS template was first fabricated by pouring PDMS prepolymer (PDMS Sylgard 184, Dow Corning Corporation, mass ratio of 10:1) onto a sandpaper template and curing it for 24 h. Next, a microstructured polyimide (PI) electrode was prepared by spin-coating PI solution (DuPont, 28% in dimethylformamide) on the PDMS template (600 r min^–1^ for 1 min) and heating the sample gradually from 40 to 240 °C (an interval of 40 °C with a duration of 0.5 h). After cooling down and peeling off the PDMS template, the PI membrance was coated with a thin layer of gold (200 nm, ion sputtering MC1000, Hitachi High-Tech Corporation) by ion sputtering. Serpentine-shaped microstructured Au-PI electrodes were cut using a laser. Flat PET electrodes were fabricated by sputtering 200 nm Au, and laser cutting, followed by spin-coating (200 r min^–1^, 2 min) an ion gel solution composed of PVDF-HFP, [EMIM] [TFSI], and acetone in a mass ratio of 1:3:9. The ion gel layer was formed after the solution evaporate at room temperature for 1 h, followed by heating at 50 °C for 2 h. The sensing units were assembled by sandwiching the iontronic layer between the microstructured Au-PI and the flat Au-PET electrode, separated and packaged using commercial PDMS (thickness: 100 μm for spacing and 50 μm for packaging). The finger-shaped hemispheres, using for softness sensing and characterization analysis, were fabricated by pouring PDMS prepolymer with a mass ratio of 10:1 into a 3D-printed mold with specially-designed serpentine bulges followed by curing at 50 °C for 5 h. The material of the finger-shaped hemispheres, using in IOP tonometer and eyelids simulation experiment, was Ecoflex 00-30 and Ecoflex 20 with a mass ratio of 1:1. The softness sensors were completed after embedding the sensing units onto the surface of the hemisphere.

**Characterization and measurement**

The microstructure of PI-Au electrode was characterized by FE-SEM, TESCAN MIRA3. The capacitance signals of the intelligent softness sensor were measured by LCR meter (E4980AL, KEYS IGHT), and the response time by TH2840B LCR meter. The multi-segment displacements control was realized by XLD-20E, Jingkong Mechanical Testing Co., Ltd. The response time and recovery time was tested at a displacement of ~1.0 mm. The humidity (52% ~ 89% RH) and temperature (40, 50 and 60 °C) were strictly controlled with a humidifier and a heating stage, respectively, in an enclosed space constructed of an acrylic box. Shore hardness samples were used to verify the softness sensory system, the relationship between Shore hardness and Young's modulus can be expressed by the following empirical equation[1]:

$E=\frac{0.0981(56+7.66S)}{0.137505(254-2.54S)}$

Where *E* is Young’s modulus (MPa), *S* is Shore hardness (HA).

**Dataset acquisition method**

For each Shore hardness sample in Fig. 3 and each object in Fig. S13, 100 sets of data were collected (Under room temperature: ~20 °C, room humidity: ~50% RH, loading speed: 5 mm min^–1^) and divided into 60 sets for training, 20 sets for validation, and 20 sets for testing. To verify the reliability and robustness of the softness sensory system, additional data were collected for each Shore hardness sample under different conditions of humidity, temperature, and loading speed, including 100 sets of data under 52% to 89% RH, 300 sets of data under 30, 40, and 50 °C, and 300 sets of data under 2.5 to 20 mm min^–1^. Three hundred sets of data for each Shore hardness sample were collected to verify the influence of eyelids thickness (Ecoflex 00-30, thickness: 0.5 mm, 1.0 mm, and 1.5 mm) on deep learning results. Similarly, 300 sets of data for each Shore hardness sample were collected to verify the effect of eyelids hardness (Ecoflex 00-30, Ecoflex 20 and PDMS) on the classification results. The displacements of softness sensing and characterization analysis is 0.2, 0.4, 0.6, 0.8, and 1.0 mm respectively. The displacements of IOP monitoring and eyelids simulation experiment is 1, 2, and 3 mm respectively.

**Deep learning method**

The softness sensing system was designed with 1DCNN neural networks. The general architecture of these networks consisted of several convolutional layers followed by pooling layers, fully connected layers, and a final output layer. Each network was trained using backpropagation and optimized with Adam optimizer. We used categorical cross entropy as our loss function. The dataset was divided into training, validation, and test sets using a stratified random sampling method (train_test_split) provided by scikit-learn Python package. For the Intraocular Pressure (IOP) dataset, which was imbalanced, we employed the Synthetic Minority Oversampling Technique (SMOTE) to the training set after splitting the data. The models were implemented using TensorFlow on an ASUS TUF-RTX3090-O24G discrete graphics card.

**Fabrication of the portable IOP tonometer**

The headset shell (RAYSHAPE) was 3D-printed with resin (Rigid 20). The displacement of the softness sensors was controlled by micro linear actuators (LAF10-024D, D-LVTTL, INSPIRE ROBOTS). The tonometer was accomplished by connecting the softness sensors, micro linear actuator and shell with several 3D-printed components. The control software was written in Python using the PyQt framework to display UI, and the mobile app was written in Swift. A home-made circuit board was used to collect data. A STM32 microcontroller with a 12-bit DAC module for generating a stable sine wave signal was used for measurement. The capacitance being measured exhibited a fixed capacitive reactance *Z* when subjected to AC excitation. A voltage signal proportional to the capacitance value output by employing a capacitive reactance-voltage conversion circuit. The voltage signal was then sampled by a 24-bit ADC, and the capacitance value was subsequently calculated by the STM32 microcontroller.

**Volunteer testing and data collecting**

Twenty-five volunteers (8 females and 17 males, 50 eyeballs) participated in our experiment, ranging in age from 20 to 60 years old. Volunteer subjects first used our portable IOP tonometer for data collection (six sets of data for each eyeball), and then used a jet measurement-based equipment (Non-Contact Tonometer NCT-200) for IOP measurement in a hospital. The portable tonometer used in this work is based on rebound measurement with a model of ICare IC100. All volunteers consented the test, and the experiment was approved by the Institution Review Board of the Southern University of Science and Technology under number 20230101.

**Note S1. Hertz contact model and calculation of Young’s modulus.**

According to the Hertz contact model, Young’s modulus can be calculated as follows:

$\text{F=}\frac{\text{4}}{\text{3}}\text{E}^{\text{*}}\text{r}^{\frac{\text{1}}{\text{2}}}\text{h}^{\frac{\text{3}}{\text{2}}}$ (S1)

$a\text{=}\sqrt{\text{rh}}$ (S2)

$\frac{\text{1}}{\text{E}^{\text{*}}}\text{=}\frac{\text{1}\text{-}{\text{v}_{\text{1}}}^{\text{2}}}{\text{E}_{\text{1}}}\text{+}\frac{\text{1}\text{-}{\text{v}_{\text{2}}}^{\text{2}}}{\text{E}_{\text{2}}}\text{ }$ (S3)

where *F* is the contact force; *a* is the radius of the contact area; *h* is the deflection of the the material under test; *v_1_* and *v_2_* are the Poisson’s ratios of the elastomer and the tested material, respectively; *E** is the effective Young’s modulus of the indentor (*E_1_*) and the objects under test (*E_2_*).

Assume that Young’s modulus of the elastomeric indentor is far higher than that of the objects (*E_1_* >> *E_2_*), Eq. S3 can be simplified as follows:

$\text{E}^{\text{*}}\text{=}\frac{\text{E}_{\text{2}}}{\text{1-}\text{v}_{\text{2}}^{\text{2}}}$ (S4)

Therefore, $E_{2}$ can be expressed as:

$\text{E}_{\text{2}}\text{=}\frac{\text{3}}{\text{4}}\left( \text{1-}\text{v}_{\text{2}}^{\text{ 2}} \right)\text{F}\text{r}^{\text{-}\frac{\text{1}}{\text{2}}}\text{h}^{\text{-}\frac{\text{3}}{\text{2}}}$ (S5)

However, in other cases, such as *E_1_*≈*E_2_*, or *E_1_* > *E_2_*, or *E_1_* < *E_2_*, Eq. S5 becomes invalid, and another method for the determination of softness is needed.

Moreover, detailed parameters including *a*, *r*, *h* and *F* are also required but the measurement of such parameters is challenging. Some other assumptions of the Hertz theory include the validation of the theory only below the elastic limit, the smaller contact area than the object, elastic half-space nature of the elastomer indentor and the object, and the frictionless contacting interface. However, these conditions can hardly be satisfied in many practical situations because most cases are non-Hertz contacts.

We thus use deep learning in combination with displacement-controlled pressure sensing to evaluation the softness of materials.

**Note S2. Data processing of the radar charts.**

Datasets of the two pressure sensors were collected at five displacements (0.2, 0.4, 0.6, 0.8, and 1.0 mm). Ten (2 × 5) channels were set for the 1DCNN model. In the test, each peak of the capacitive signals was normalized (*C_x_/C_max_*, *x* = 0.2, 0.4, 0.6, 0.8, and 1, where *C_max_* is the maximum peak value). For example, ten characteristic peaks were generated when the indentor contacted a specimen (87 HA) at five different displacements. In this case, *C_max_* is *C_1_* of sensor #1; and we used *C_0.2_/C_1_* of sensor #1 for channel 1 and *C_0.2_* of sensor #2 */C_1_* of sensor #1 for channel 6.

**Note S3. Calculation of kappa coefficient.**

A confusion matrix was given as an example as follows:

| Label | 1 | 2 | 3 |
| --- | --- | --- | --- |
| 1 | a | b | c |
| 2 | d | e | f |
| 3 | g | h | i |

The calculation is as follows:

$\text{P}_{\text{0}}\text{=}\frac{\text{a+e+i}}{\text{a+b+c+d+e+f+g+h+i}}$ (S6)

$\text{P}_{\text{e}}\text{=}\frac{\left( \text{a+d+g} \right)\text{×}\left( \text{a+b+c} \right)\text{+}\left( \text{b+e+h} \right)\text{×}\left( \text{d+e+f} \right)\text{+(c+f+i)×(g+h+i)}}{\text{(a+b+c+d+e+f+g+h+i)}^{\text{2}}}$ (S7)

$\text{Kappa=}\frac{\text{P}_{\text{0}}\text{-}\text{P}_{\text{e}}}{\text{1-}\text{P}_{\text{e}}}$ (S8)


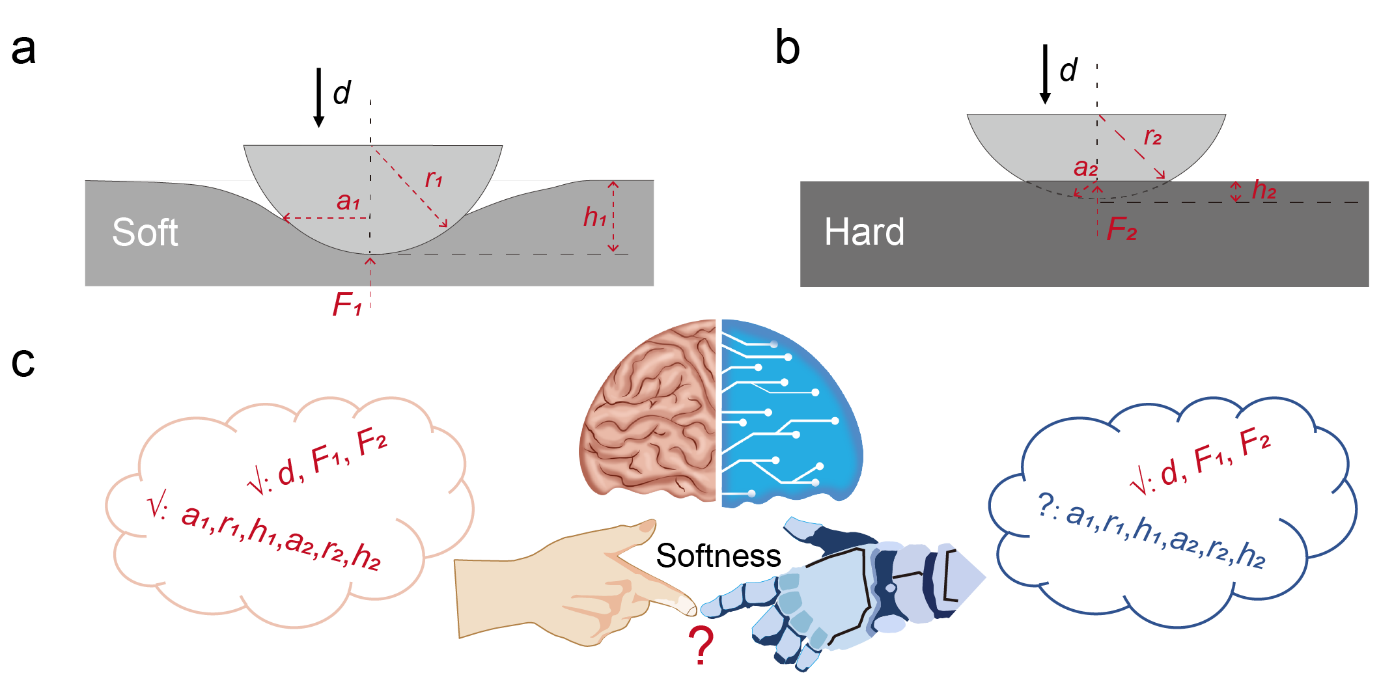


**Figure S1.** Schematics of the contact between a spheric soft indentor and an object under detection. (a) Soft contact. (b) Hard contact. (c) Softness is a concept determined by both force and geometric parameters.


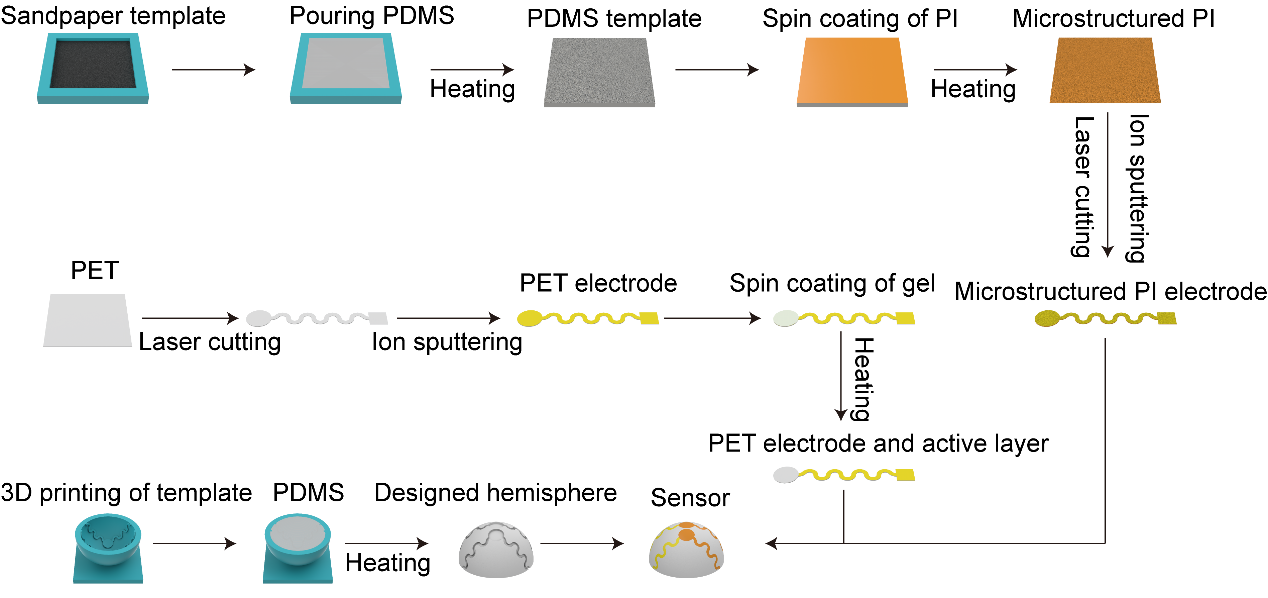


**Figure S2.** Fabrication of the indentor and the integration of sensors with the indentor.


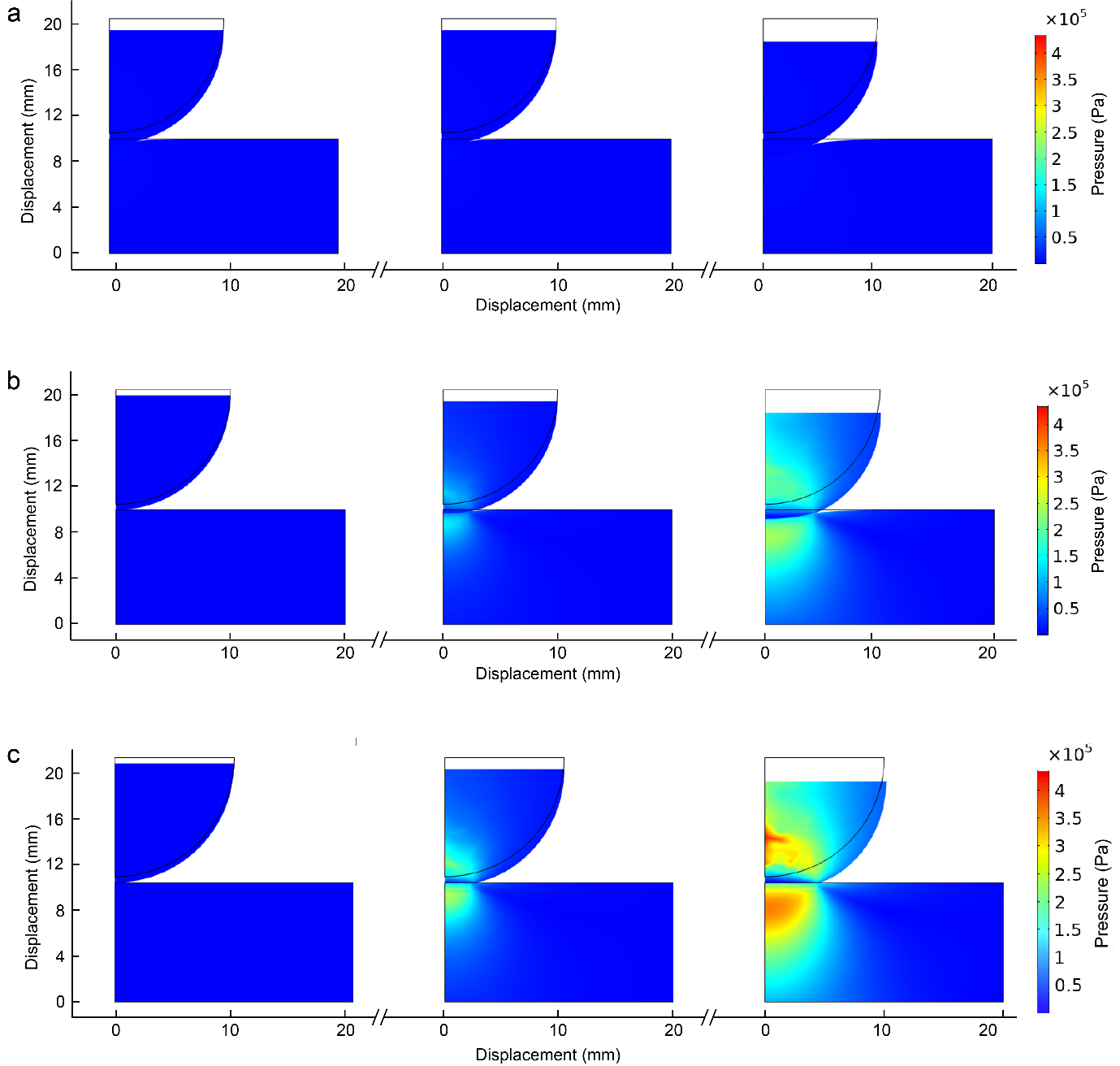


**Figure S3.** Finite element analysis of the contact between an elastomeric (PDMS) indentor and tested objects with different Young’s moduli. Moduli of the materials are: (a) 37 kPa; (b) 2.6 MPa; (c) 200 GPa.


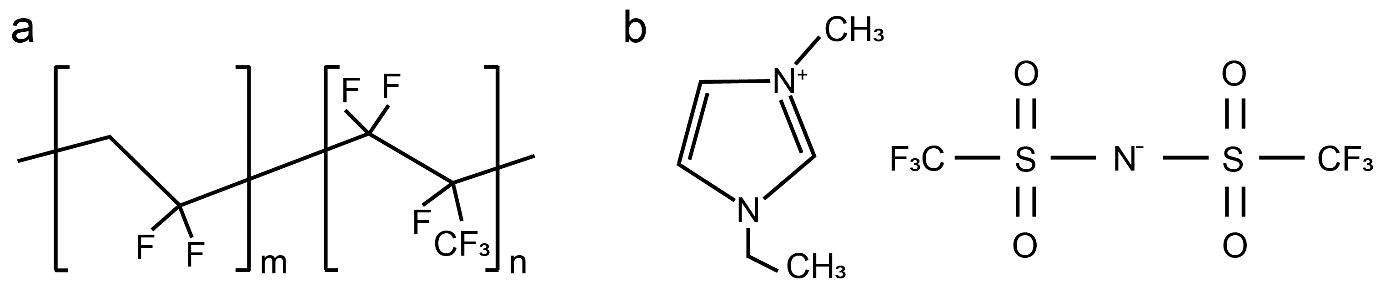


**Figure S4.** Chemical structure for the components of the ionic gel. (a) PVDF-HFP. (b) [EMIM] [TFSI].


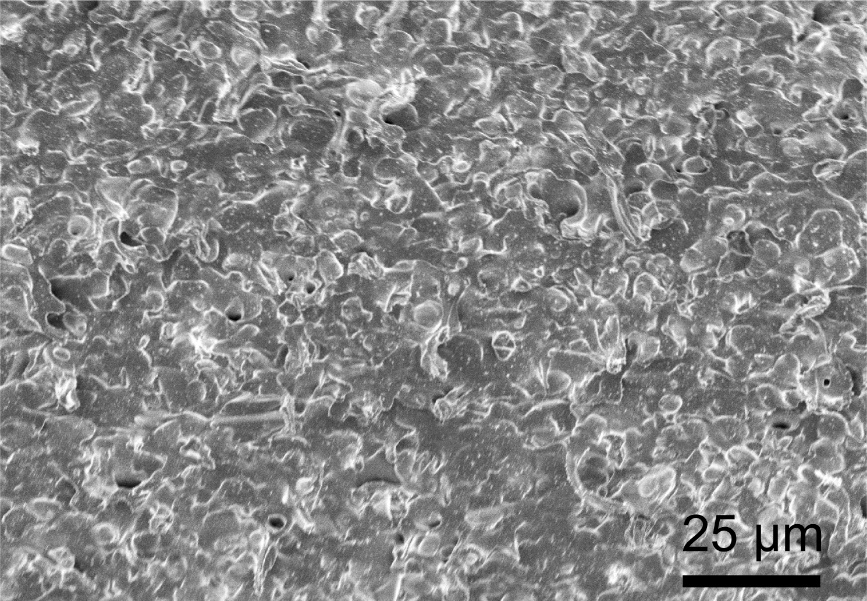


**Figure S5.** Scanning electron microscopy (SEM) image of the PI electrode with microstructures.


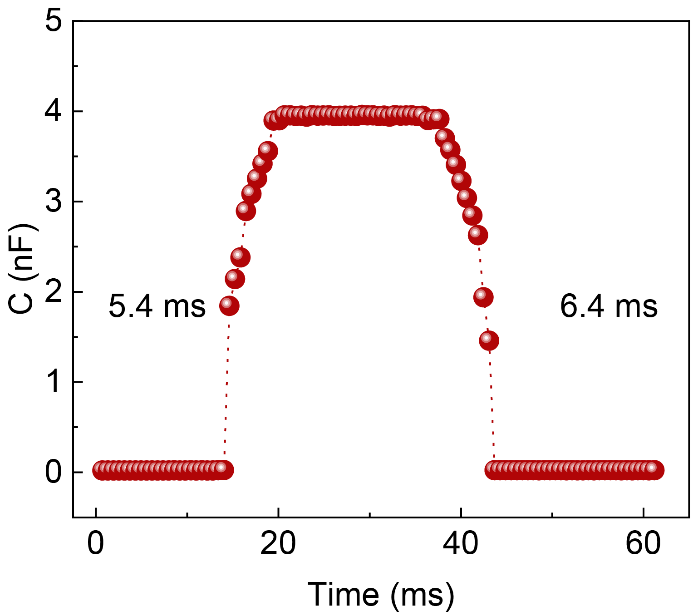


**Figure S6.** Response-relaxation time of the flexible pressure sensor.


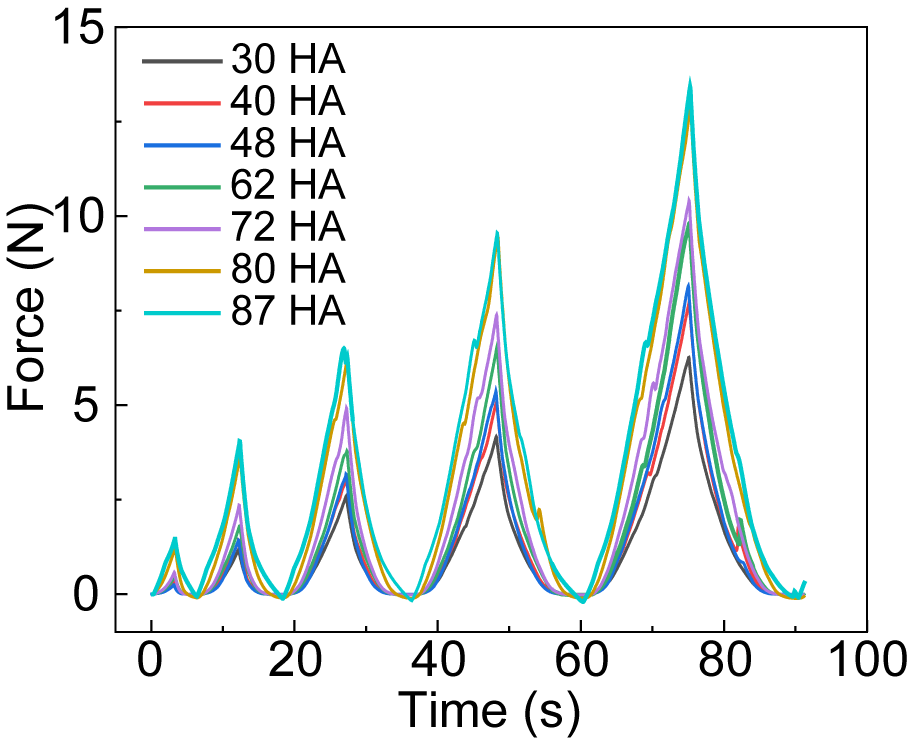


**Figure S7.** Force signals of specimens with different Shore hardness values measured using the indentor.


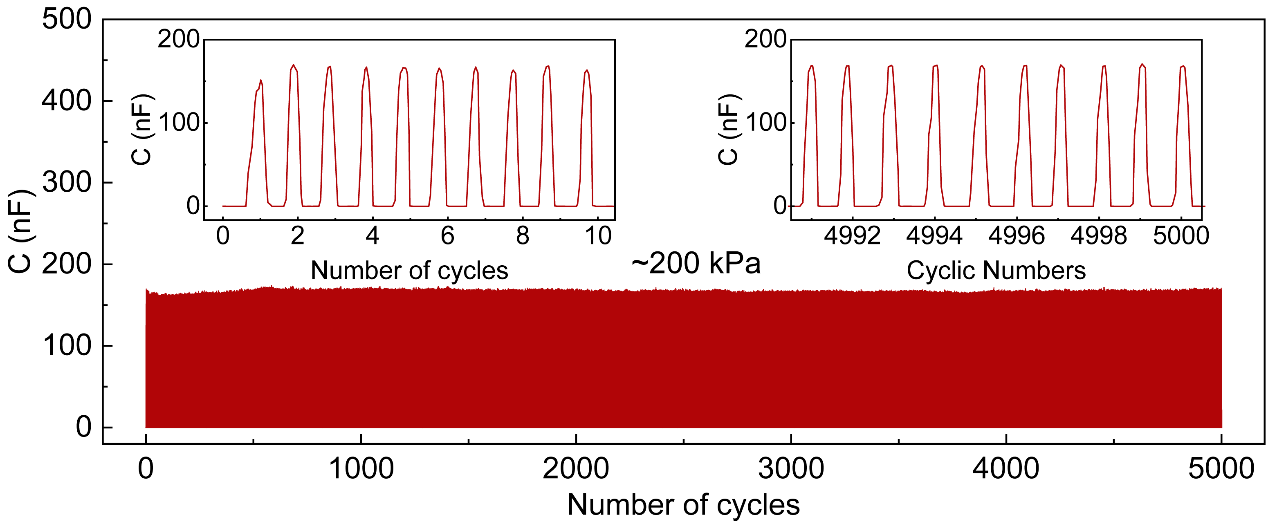


**Figure S8.** Capacitance signal of a pressure sensor loaded-unloaded over 5,000 cycles under a peak pressure of 200 kPa. The result indicates high stability over cycling.


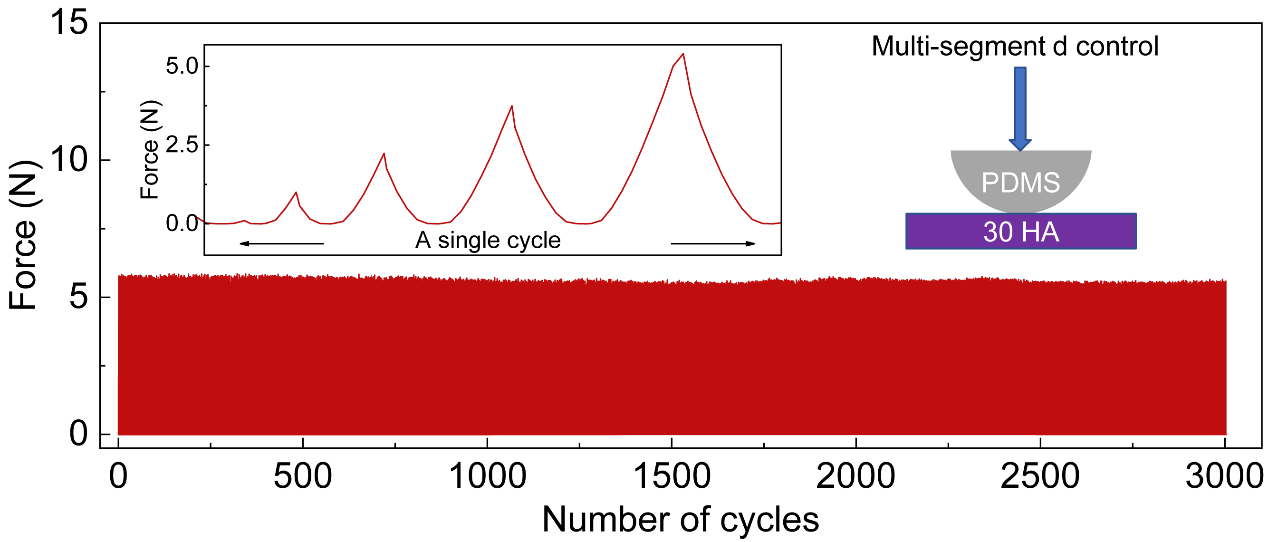


**Figure S9.** Working stability of the PDMS indentor over 3,000 cycles of loading-unloading under a multi-segment displacement control.


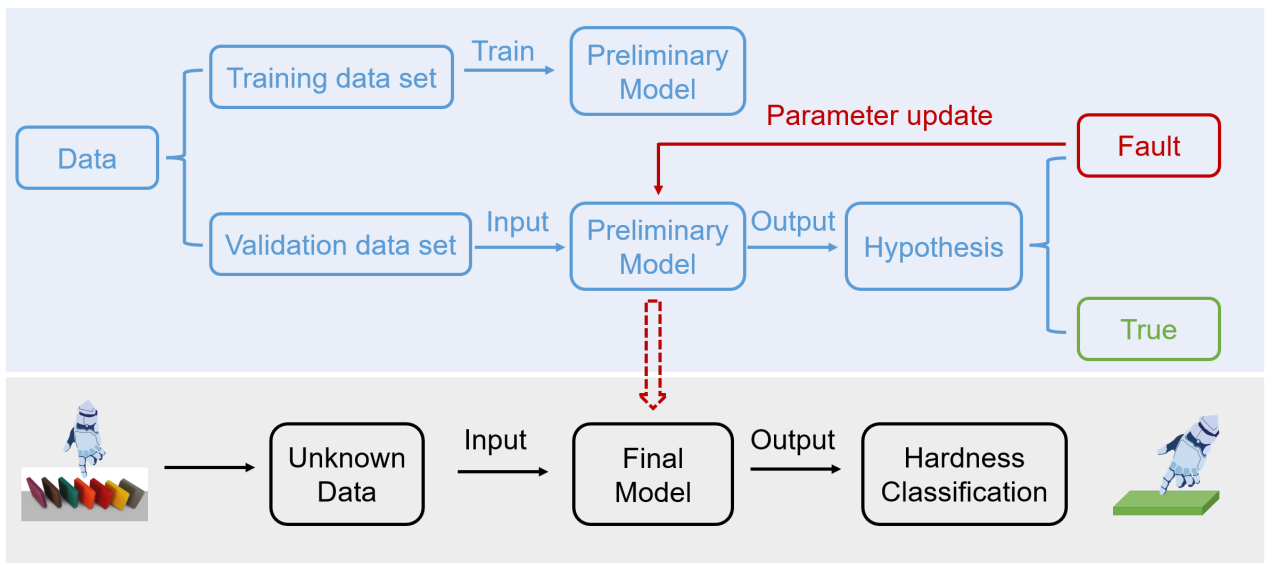


**Figure S10.** Working principle of the deep learning model for the sensory system.


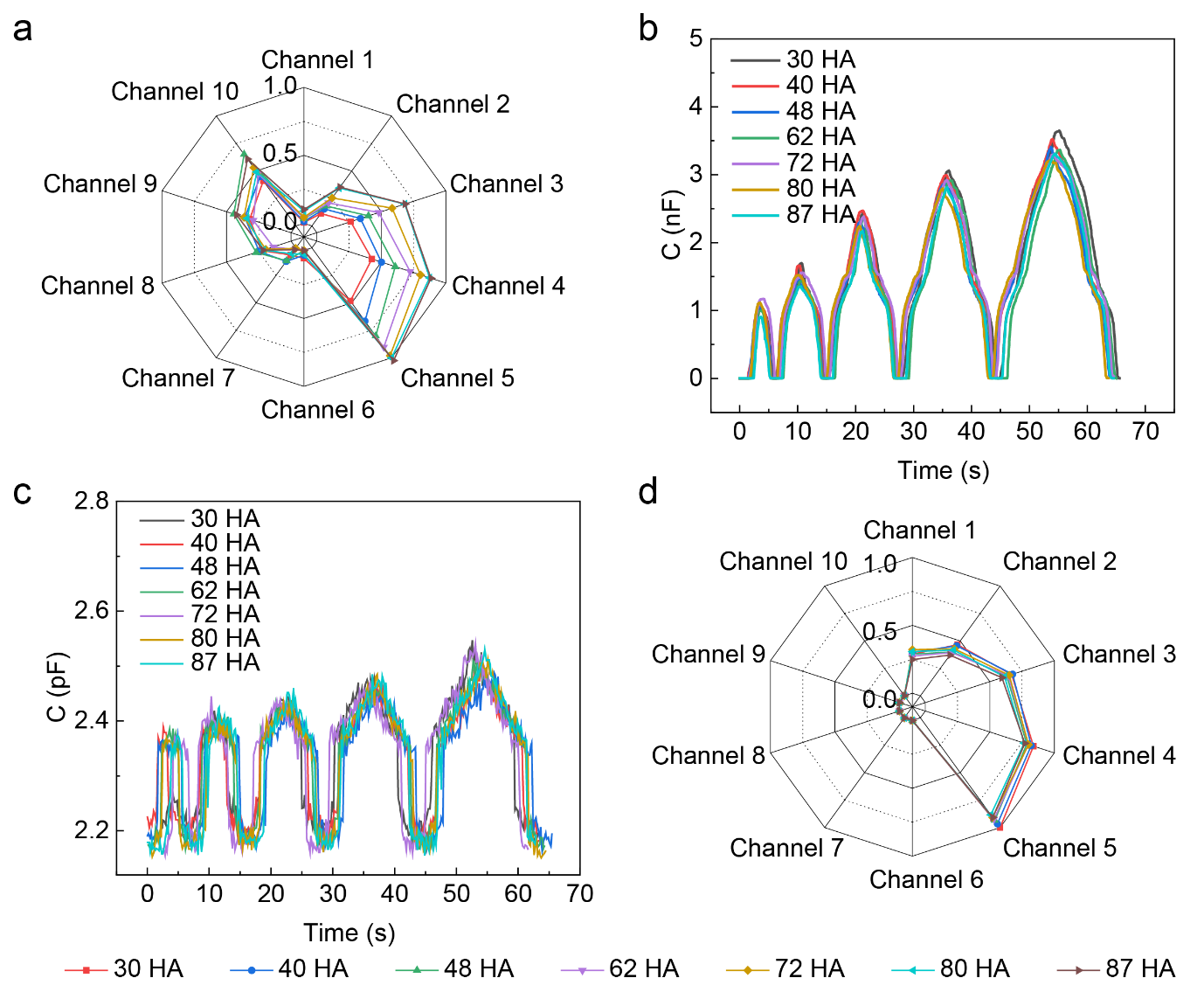


**Figure S11.** Performance of a sensory system based on force control. (a) Radar chart of the sensory system based on displacement control. Signals of (b) sensor #1 and (c) sensor #2 by contacting objects with different softness values under a multi-segment force control. (d) Radar chart of a sensory system based on force control.


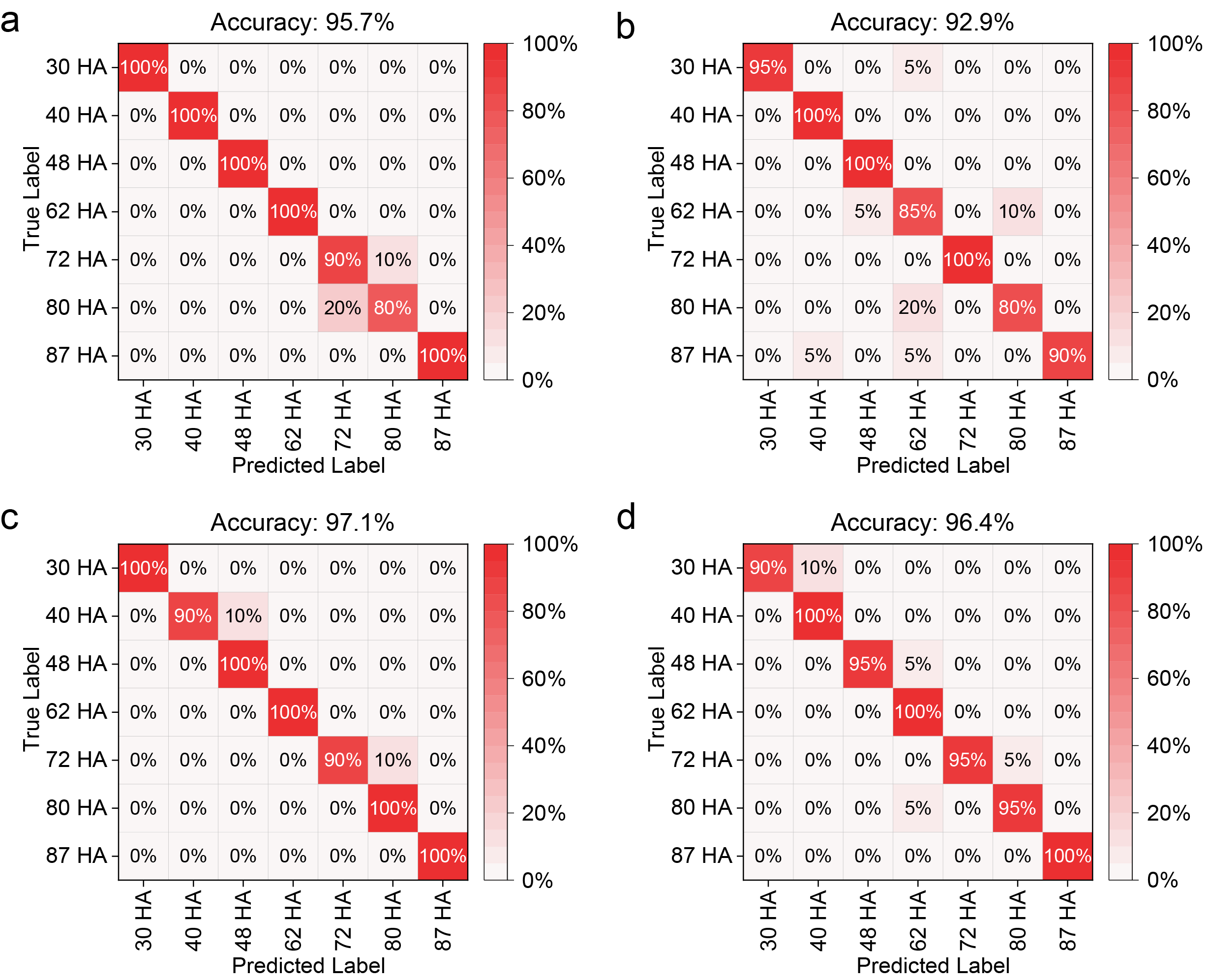


**Figure S12.** Confusion matrixes of different datasets collected at different conditions. (a) Data of using only sensor #1. (b) Data of using only sensor #2. (c) Data of using four displacements (0.2, 0.4, 0.6 and 0.8 mm). (d) Data of using a single displacement (1 mm).


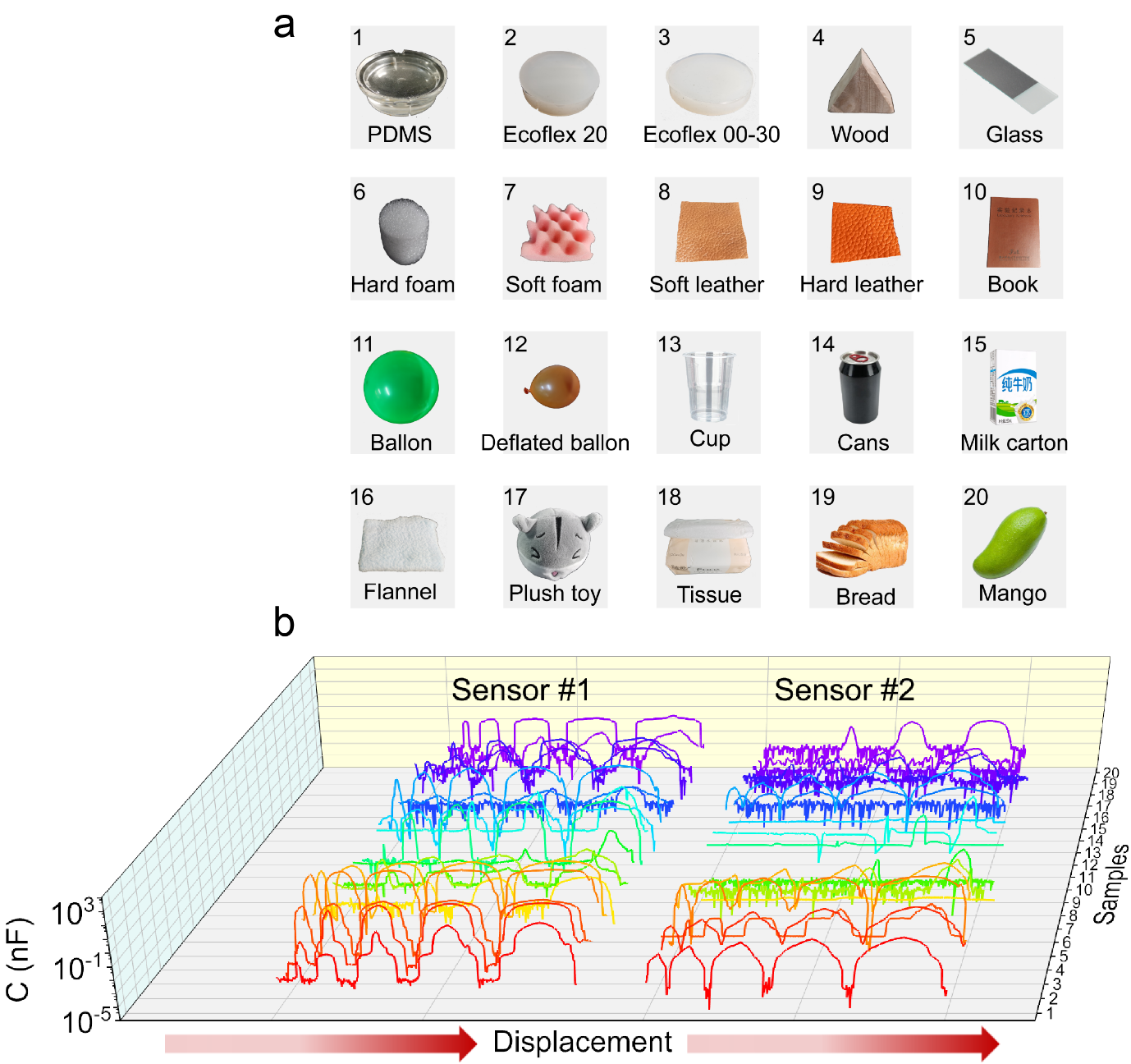


**Figure S13.** Robotic haptic application of the sensory system. (a) Twenty objects under testing. (b) Sensing signals of the 20 objects.


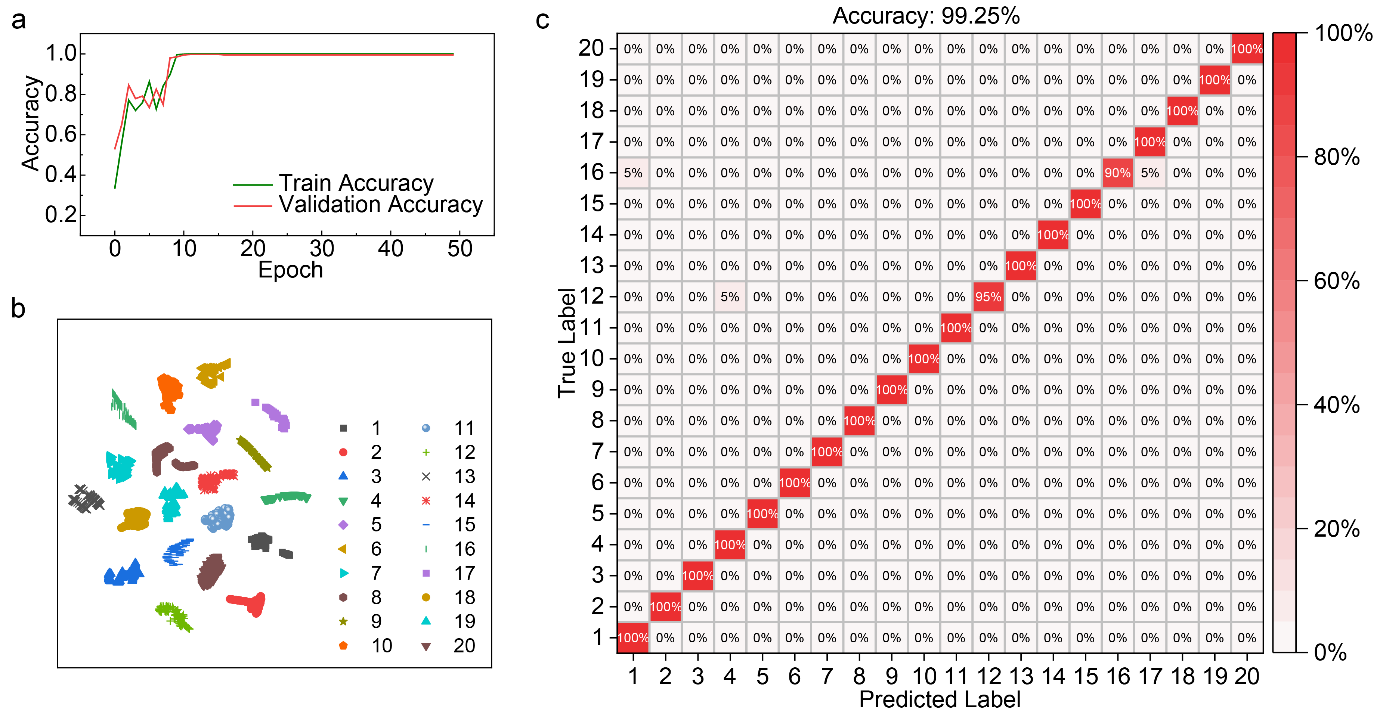


**Figure S14.** Results of the deep learning model for robotic haptic application. (a) Accuracy as a function of the number of epochs. (b) The t-SNE visualization of clustered data of the twenty objects. (c) Confusion matrix showing that the classification accuracy is 99.25%.


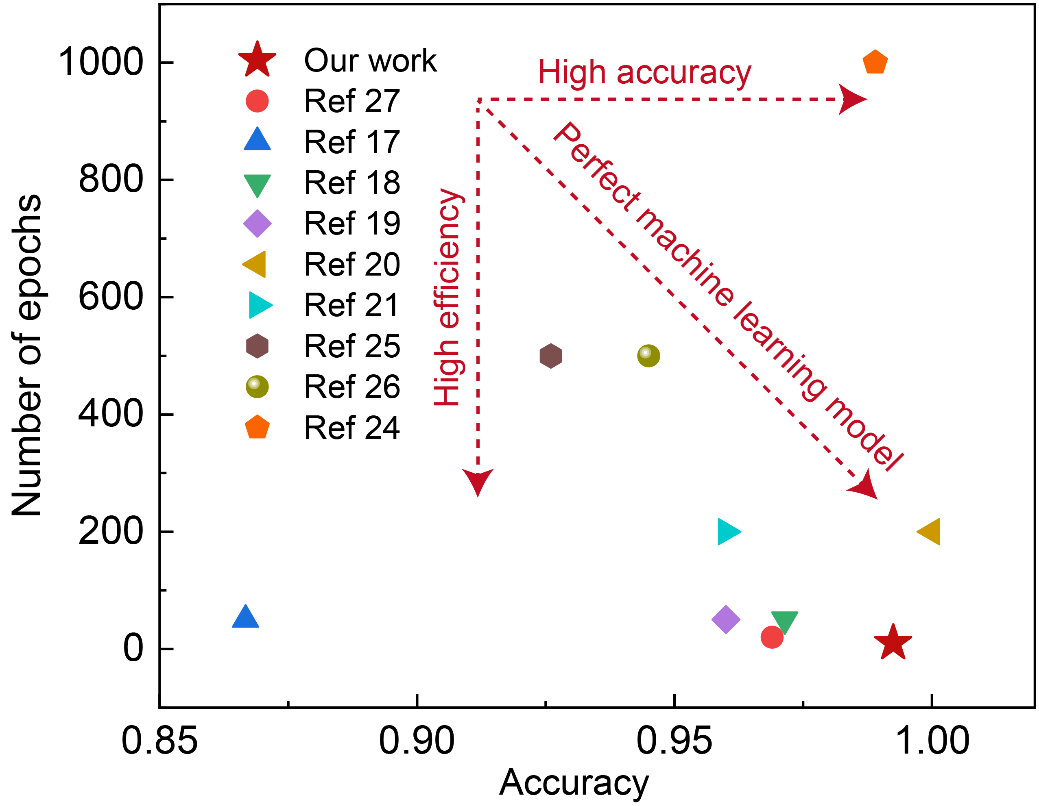


**Figure S15.** Accuracy and efficiency of our sensory system and its comparison with that of existing work. Our system achieves a high accuracy in only a few epochs, indicating a higher efficiency of the system.


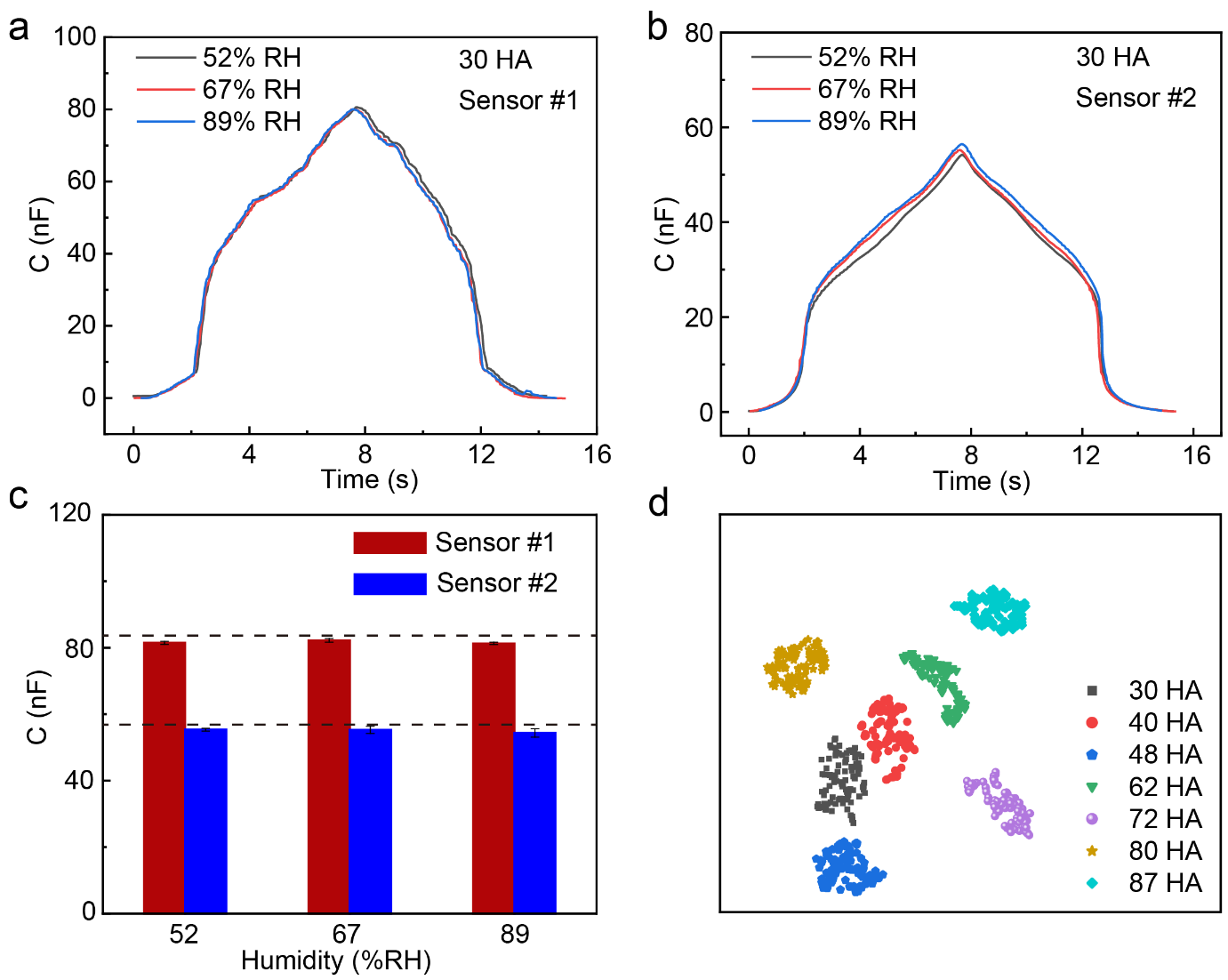


**Figure S16.** Effect of humidity on the performance of the sensory system. Signals of sensor #1 (a) and sensor #2 (b) by contacting an object of 30 HA under different humidities. (c) Peak capacitance values of sensor #1 and sensor #2. (d) The t-SNE result.

**
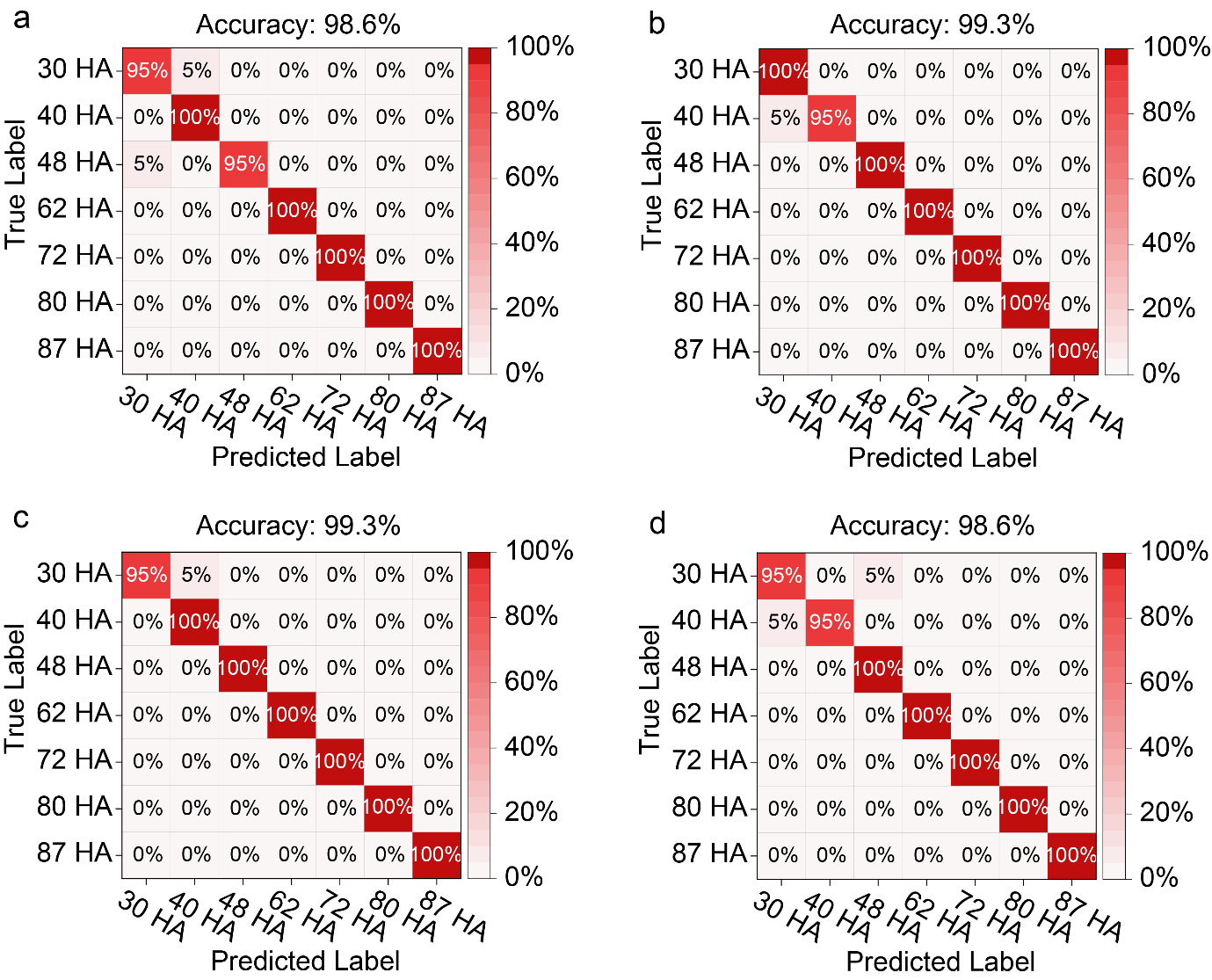
Figure S17.** Confusion matrixes of the test at different humidities. (a) 52% RH. (b) 67% RH. (c) 89% RH. (d) Random humidities.


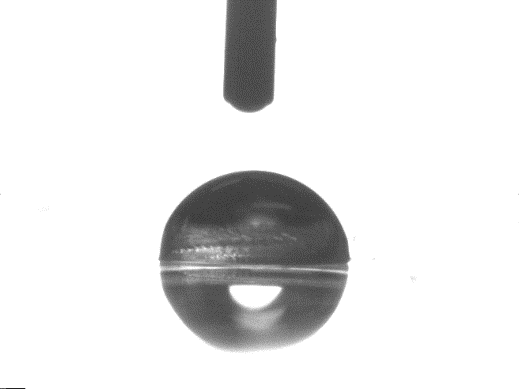


**Figure S18.** Water contact angle of the ionic material is determined to be 91°.


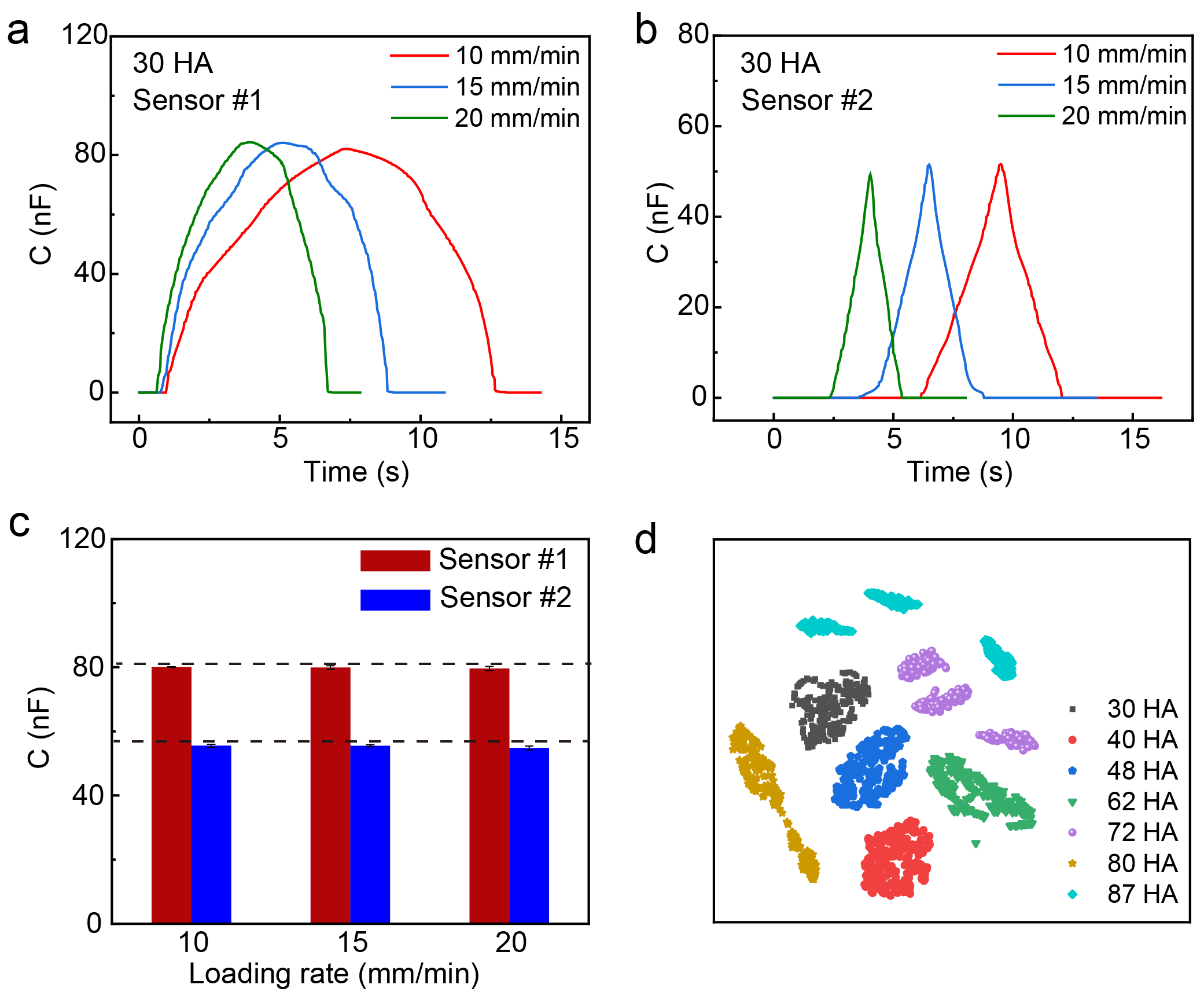


**Figure S19.** Effect of loading rate on the response of the sensors and the performance of the sensory system. (a, b) Signals of sensor #1 and sensor #2 when interacting with an object of 30 HA under different loading rates. (c) Peak capacitance values of signals from sensor #1 and sensor #2. (d) The t-SNE result.


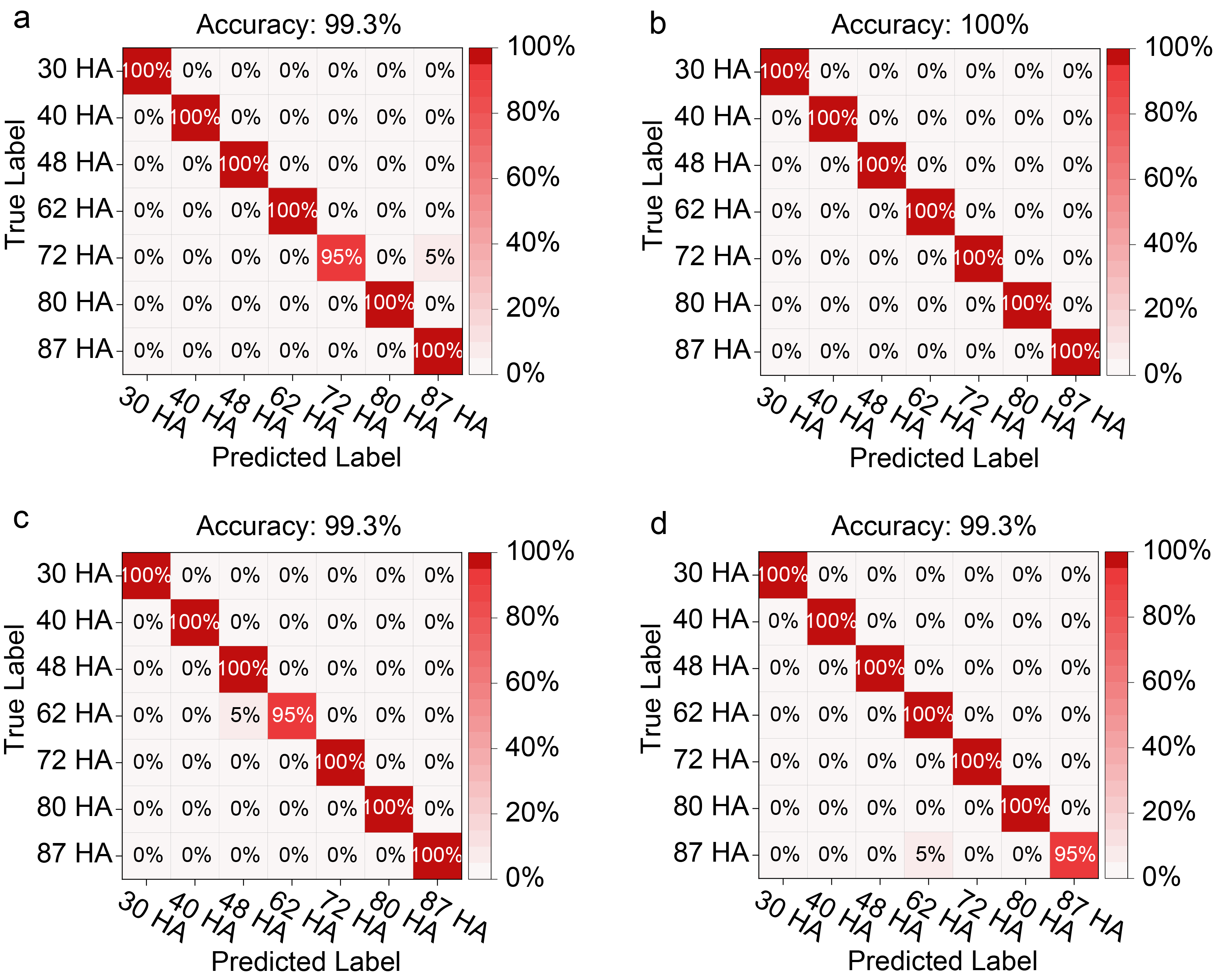


**Figure S20.** Confusion matrixes for the classification of materials with different Shore Hardness values at different loading rates. (a-c) Loading rate: 10, 15, and 20 mm min^-1^, respectively. (d) Random loading rate.

**
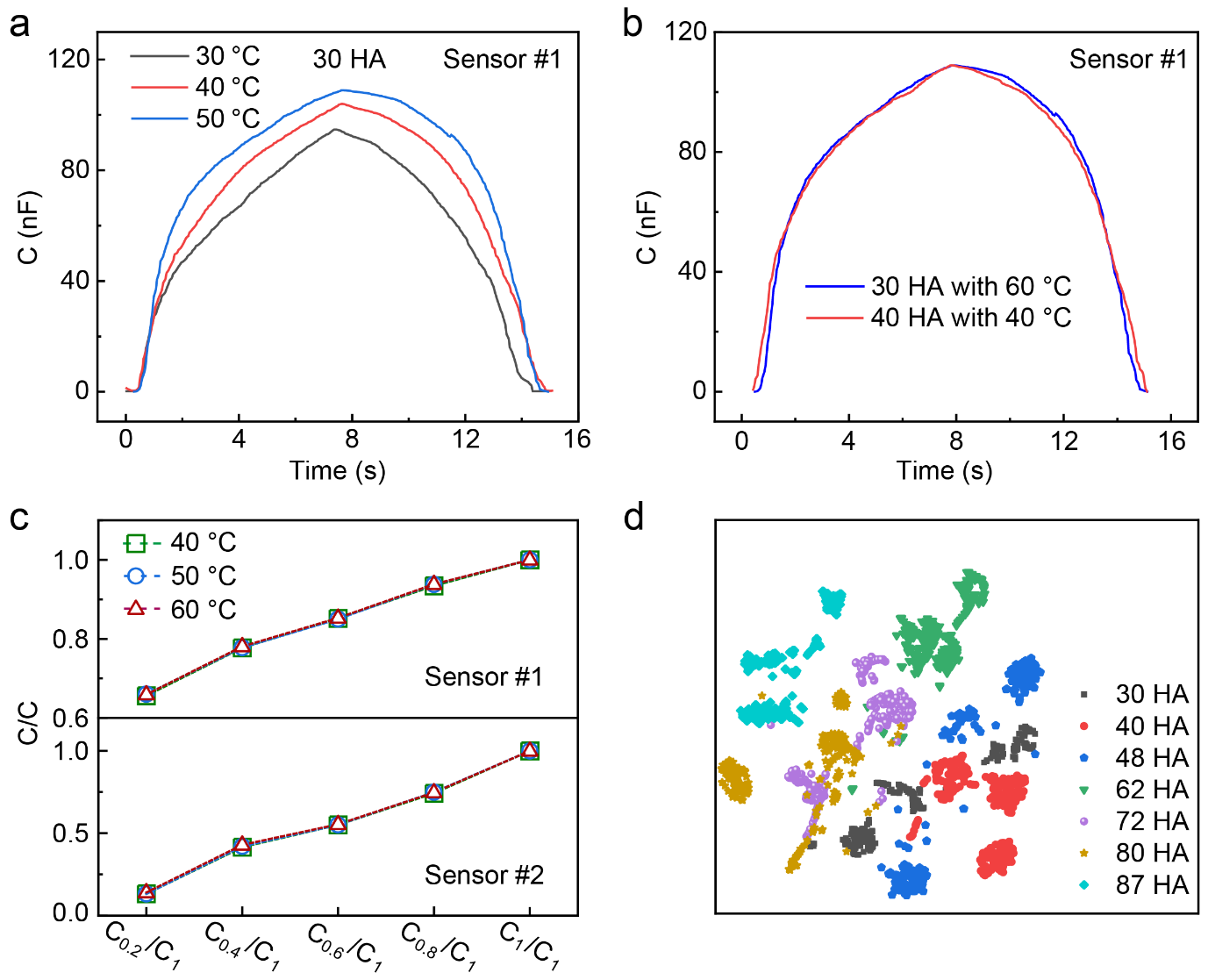
Figure S21.** Effect of working temperature on sensing signals and the performance of the system. (a) Signals of sensor #1 when contacting an object with a Shore hardness value of 30 under different temperatures of 30, 40, and 50 °C. (b) Signals of sensor #1 when touching a material of Shore hardness value of 30 at 60 °C, and that by touching a material of Shore hardness value of 40 at 40 °C. (c) Normalized peak capacitance values at different displacements and different temperatures. (d) Corresponding t-SNE result.

**
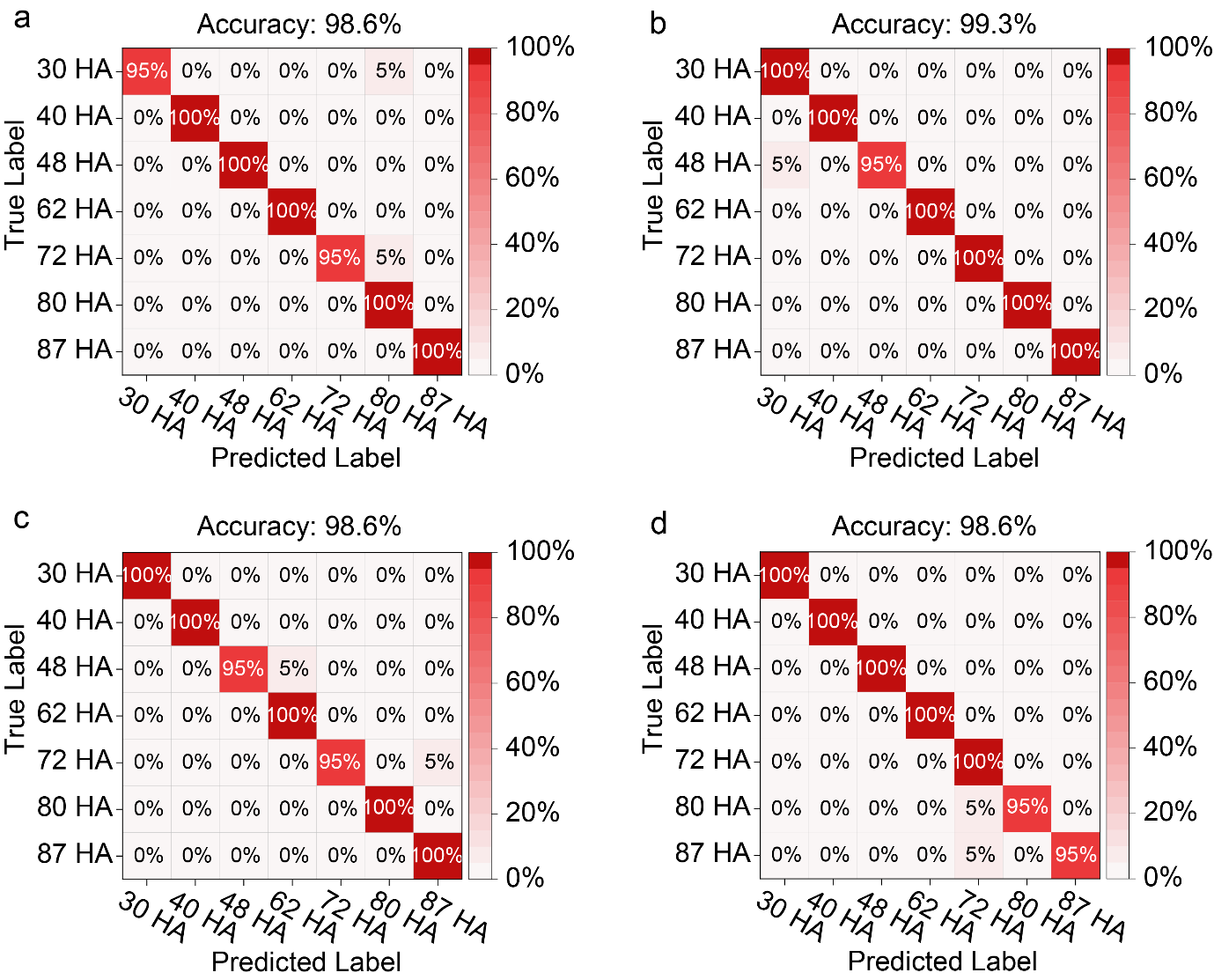
Figure S22.** Confusion matrixes of the system at different temperatures. (a) 40 °C. (b) 50 °C. (c) 60 °C. (d) Mixed temperatures.


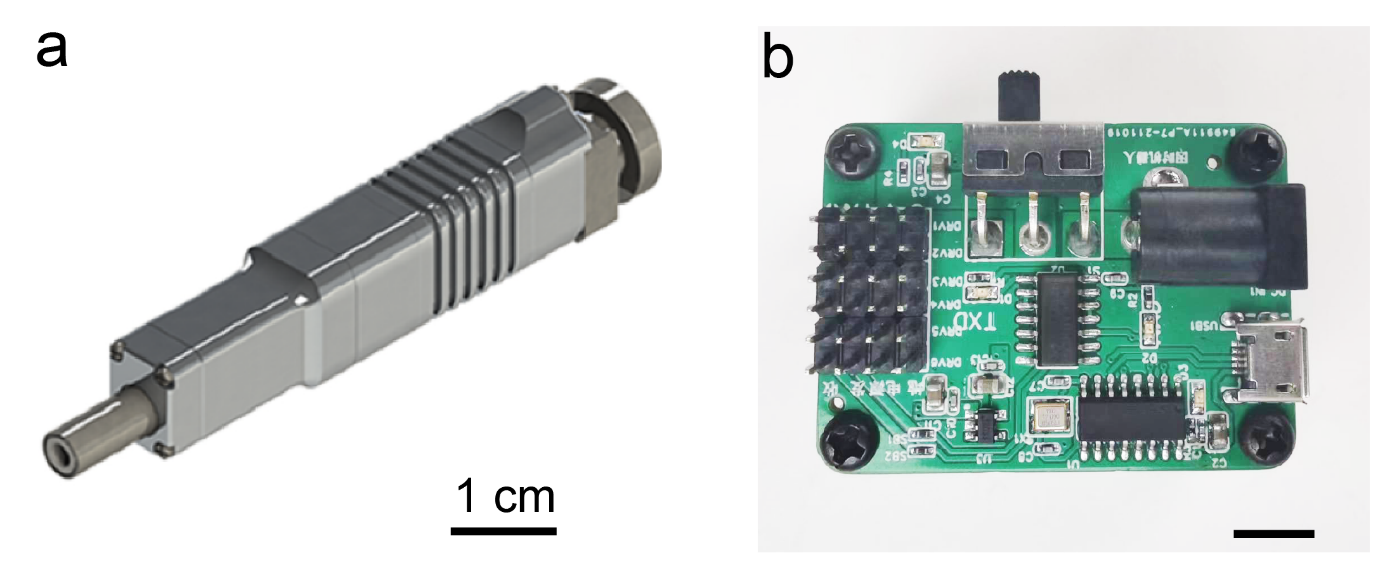


**Figure S23.** System for displacement control. (a) The micro linear actuator. (b) Circuit board.


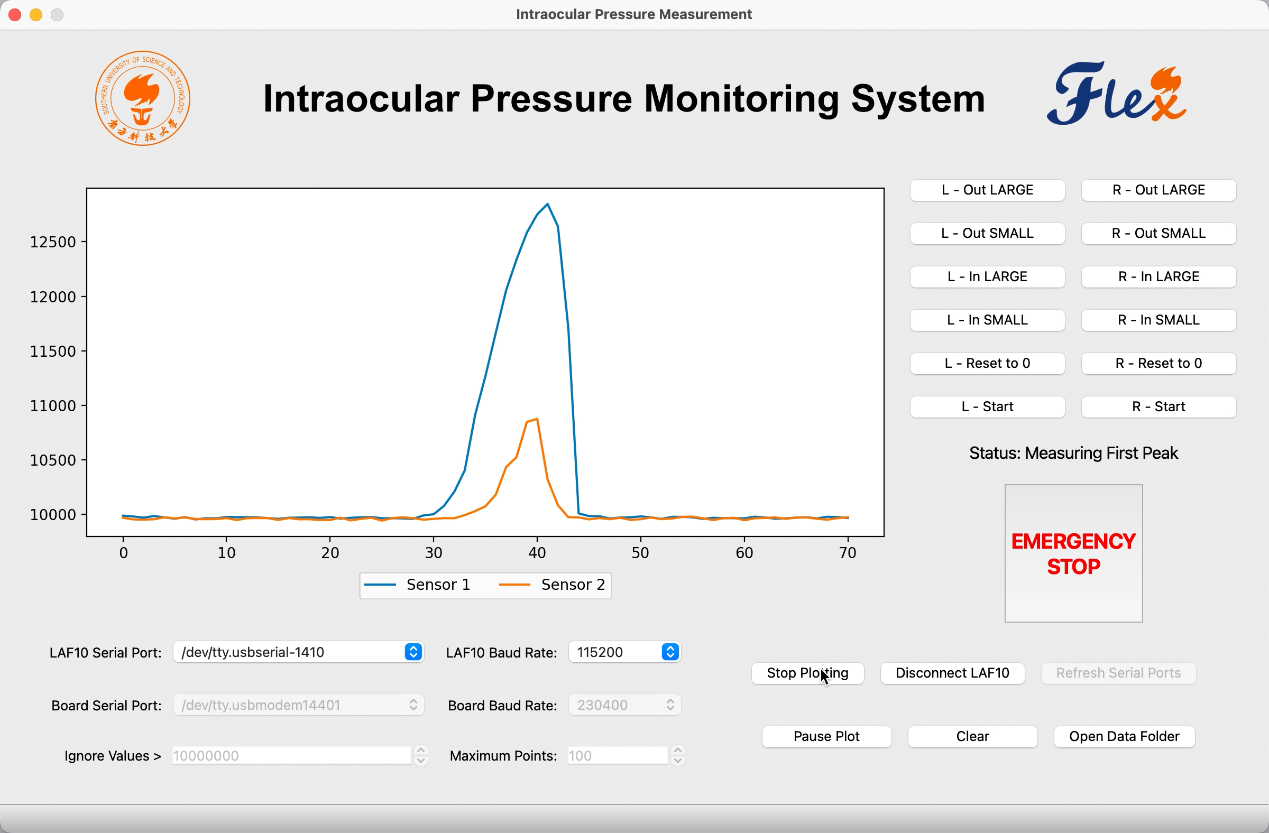


**Figure S24.** Interface of the portable IOP tonometer.


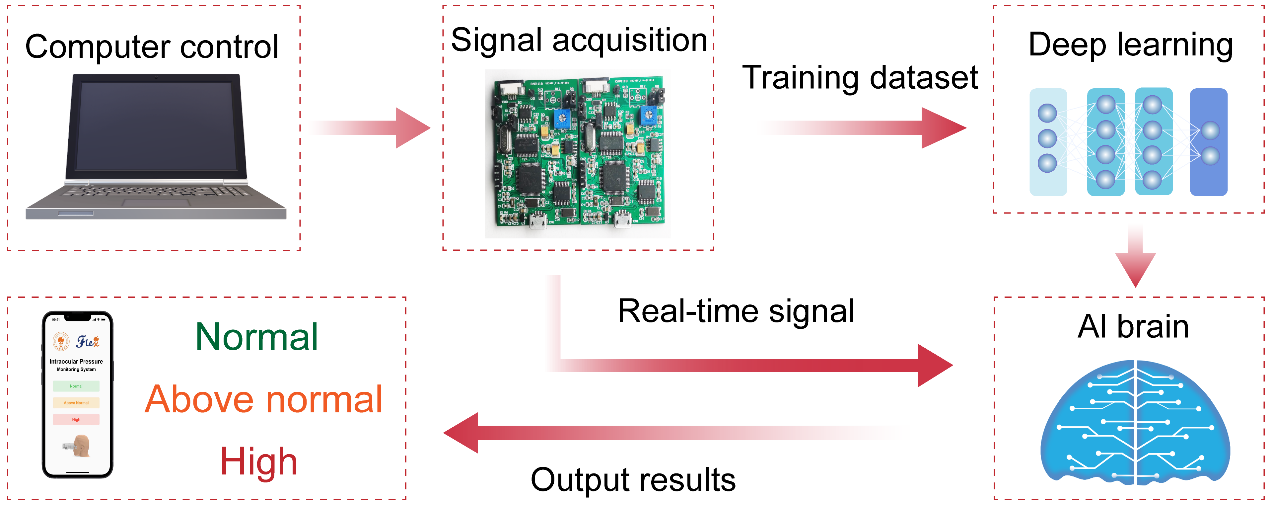


**Figure. S25.** Work flow chart of the portable IOP tonometer.


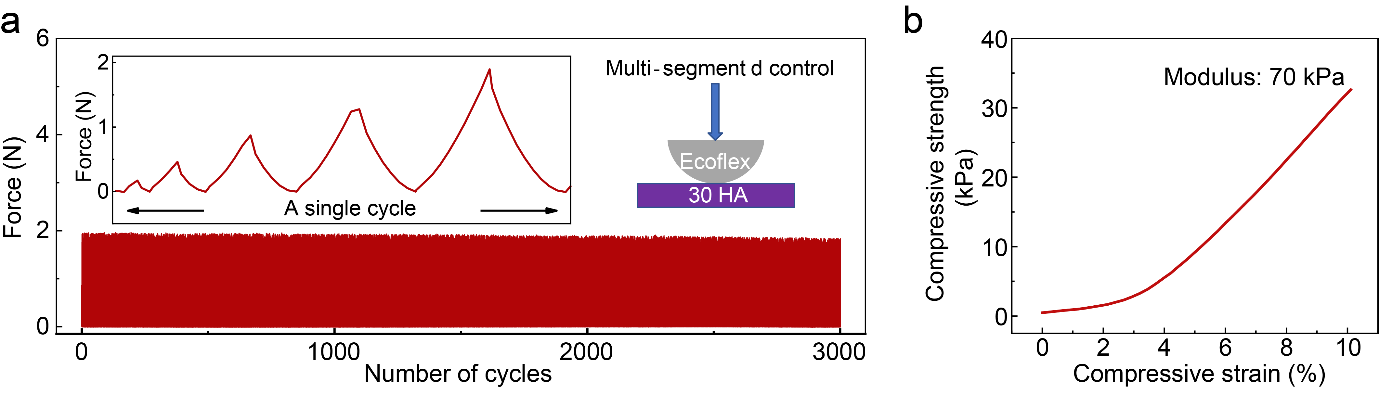


**Figure S26.** Mechanical performances of the elastomer for the hemispheric indentor. (a) Working stability of the elastomeric indentor working at a displacement-control mode over 3000 cycles. (b) Young’s modulus of the material used for the soft indentor. The material is a mixture of Ecoflex 00-20 and Ecoflex 00-30 at a weight ratio of 1:1.


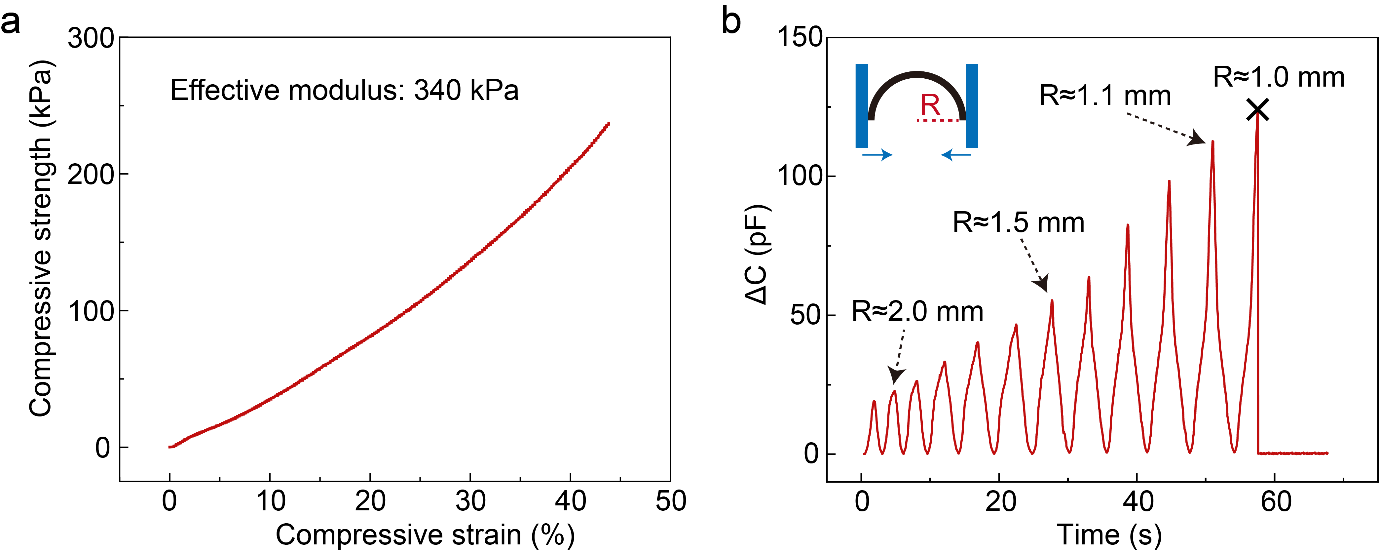


**Figure S27.** The flexibility of the pressure sensor. (a) Effective modulus: 340 kPa. (b) The minimum radius of curvature for bending: 1.1 mm.


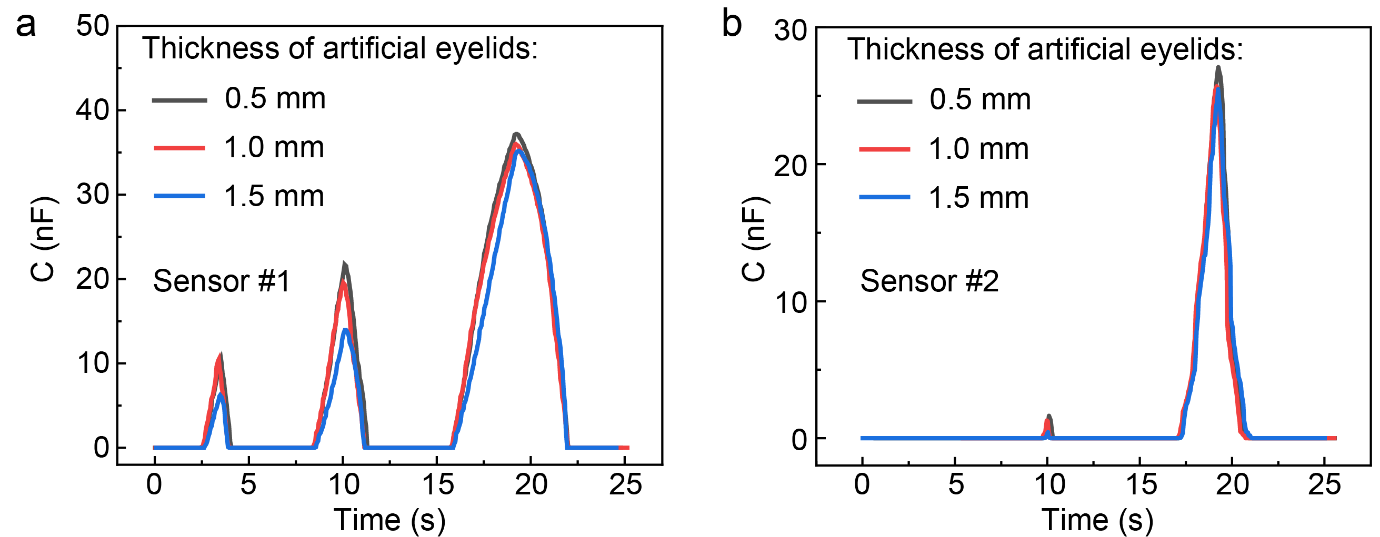


**Figure S28.** Signals of (a) sensor #1 and (b) sensor #2 under different thicknesses of artificial eyelid (displacement: 1, 2, and 3 mm).


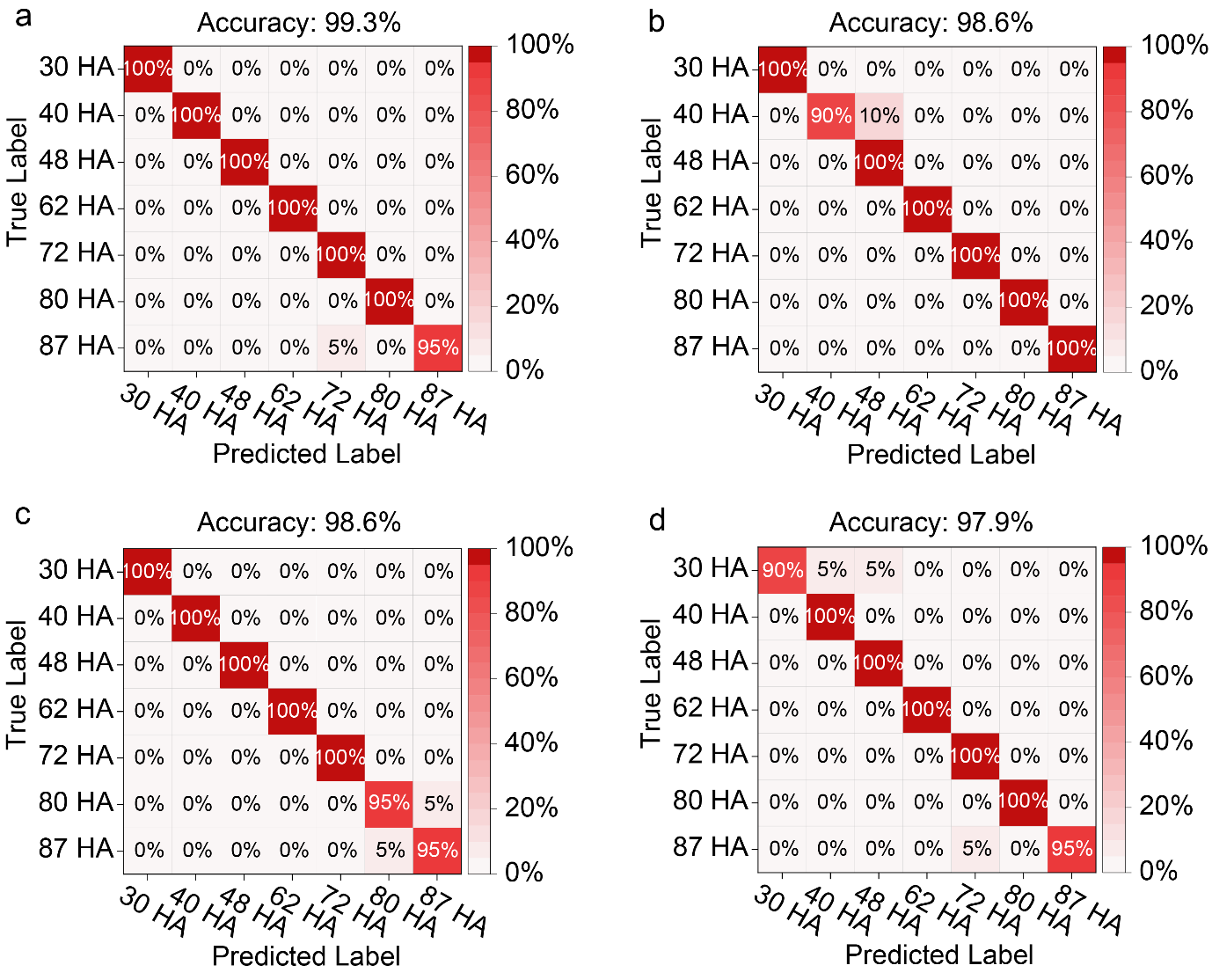


**Figure S29.** Confusion matrixes at different thicknesses of the artificial eyelid. (a) 0.5 mm. (b) 1.0 mm. (c) 1.5 mm. (d) Mixed thicknesses.


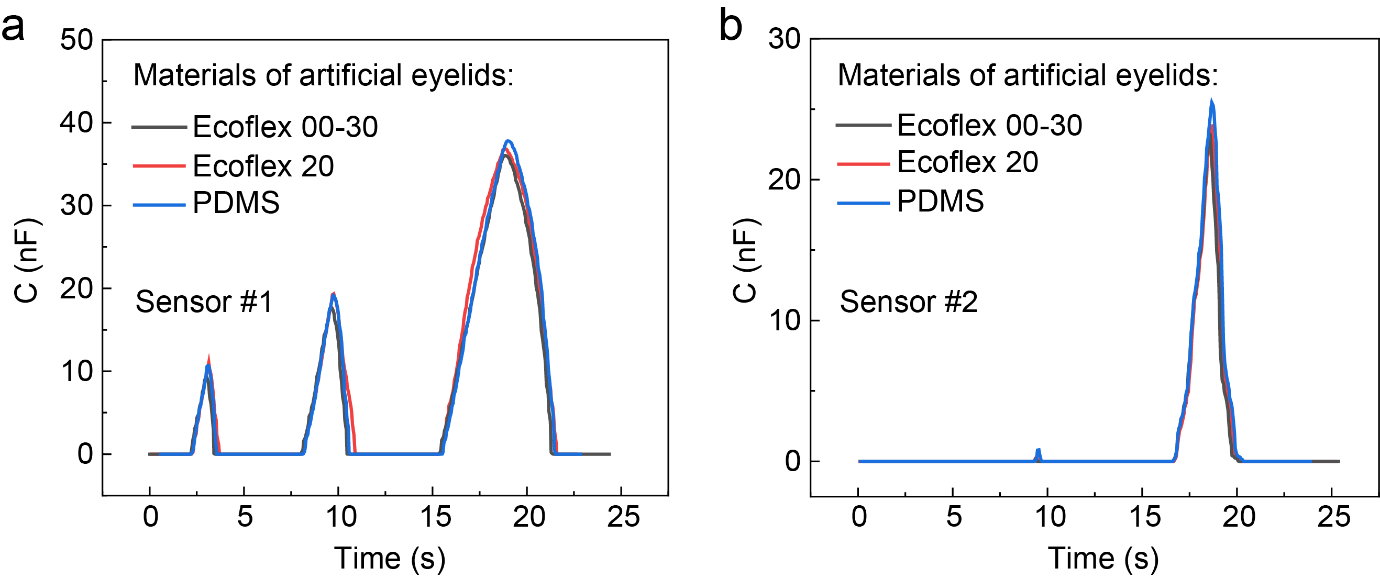


**Figure S30.** Signals of (a) sensor #1 and (b) sensor #2 when using different materials (Ecoflex 00-30, Ecoflex 20, PDMS) as the artificial eyelid.


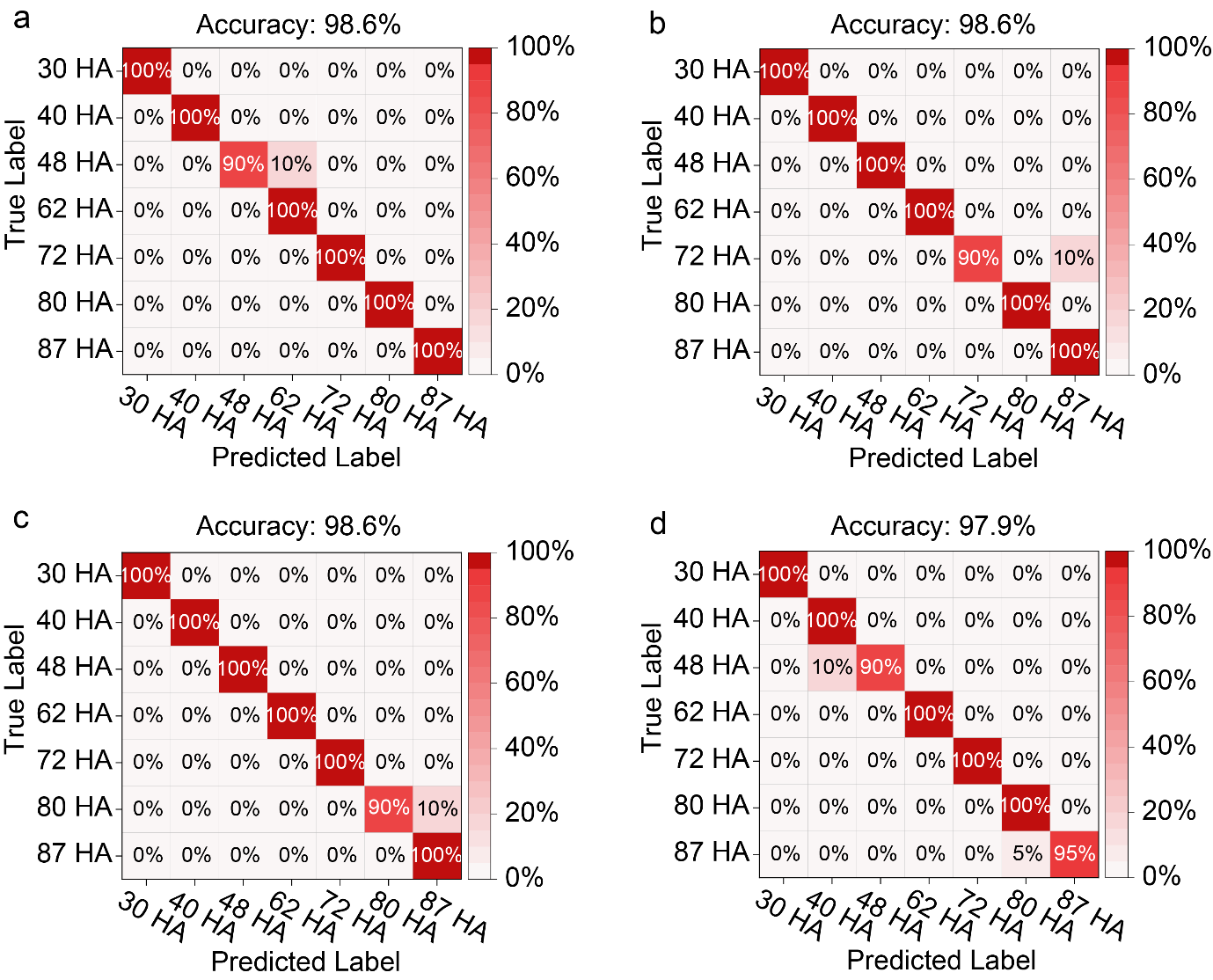


**Figure S31.** Confusion matrixes at different thicknesses of the artificial eyelid. (a) Ecoflex 00-30. (b) Ecoflex 20. (c) PDMS. (d) Mixed materials.


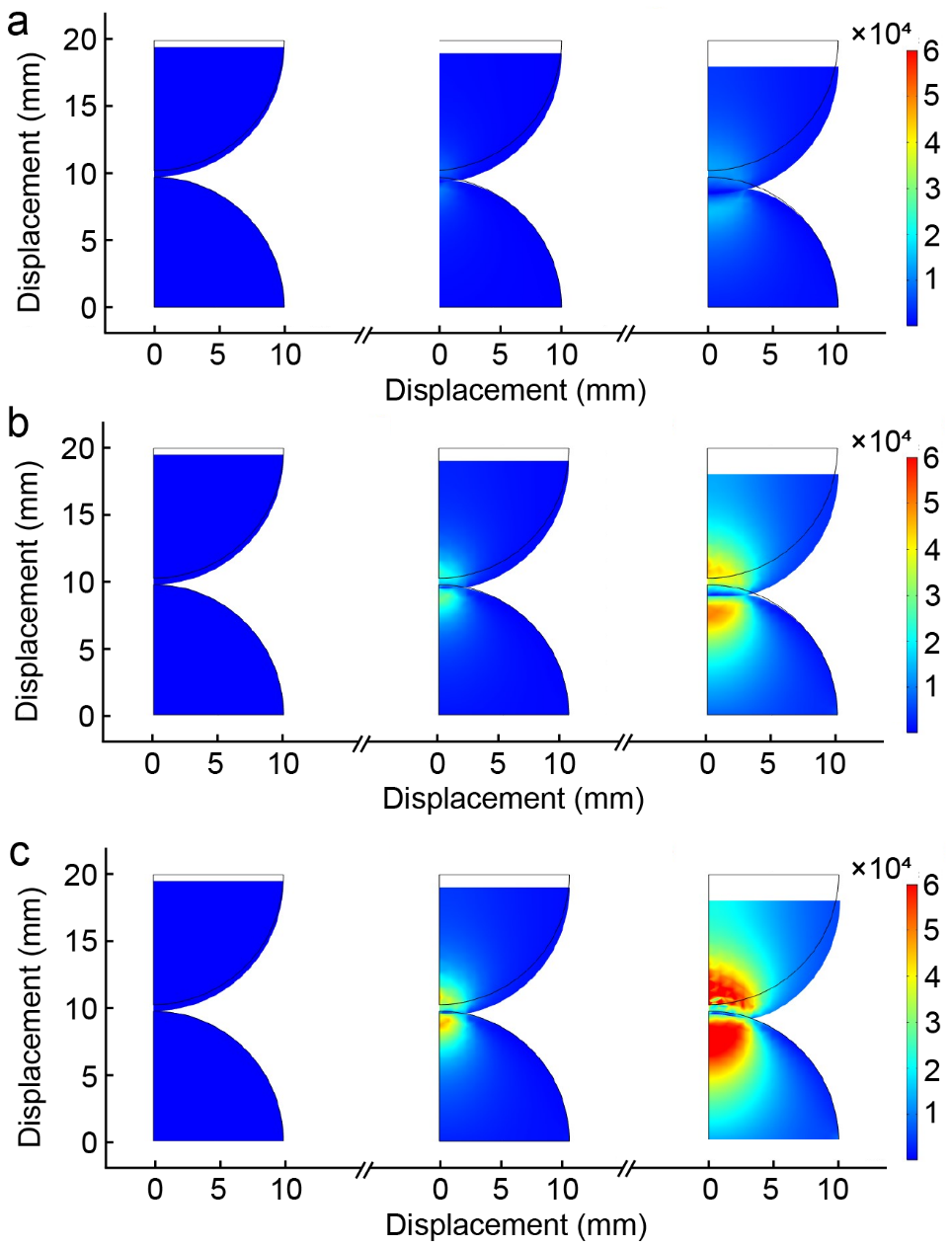


**Figure S32.** Simulation of the contact between an elastomeric hemisphere and a hemispheric object. The modulus of the elastomeric hemisphere is 0.5 MPa, and the moduli of the contacted hemispheres are (a) 0.01 MPa, (b) 0.5 MPa, and (c) 10 MPa.


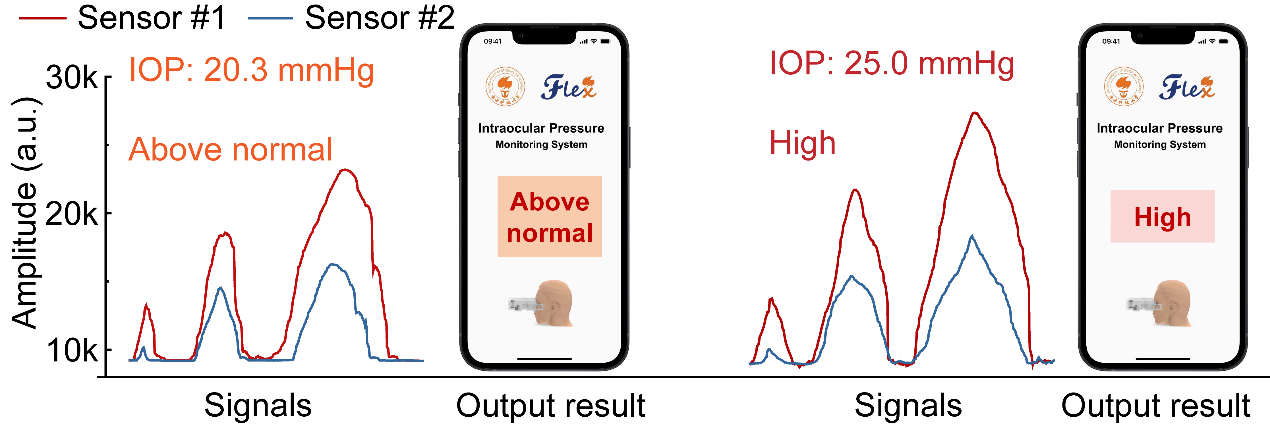


**Figure S33.** Examples of capacitance signals for the two sensors and corresponding IOP results assessed using our tonometer. The signals were collected from two volunteer subjects.

**
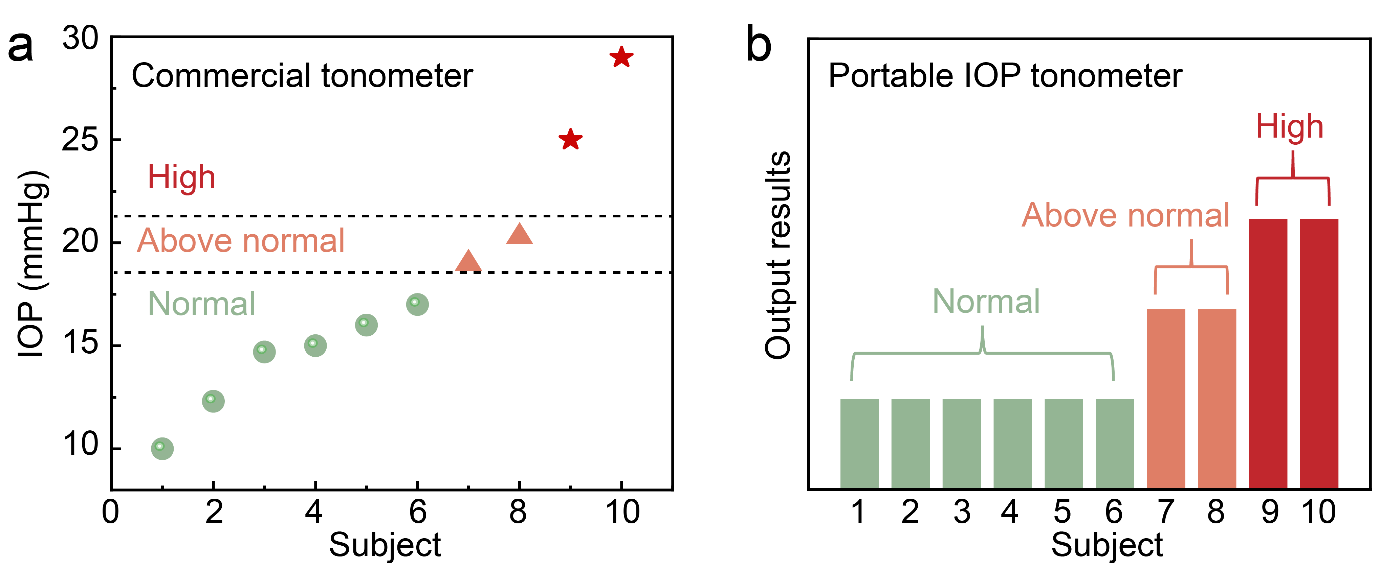
Figure S34.** Comparison of our tonometer with a commercial tonometer. (a) Results of 10 subjects tested using a jet measurement-based IOP tonometer (Non-Contact Tonometer NCT-200). (b) IOP results measured using our portable IOP tonometer.


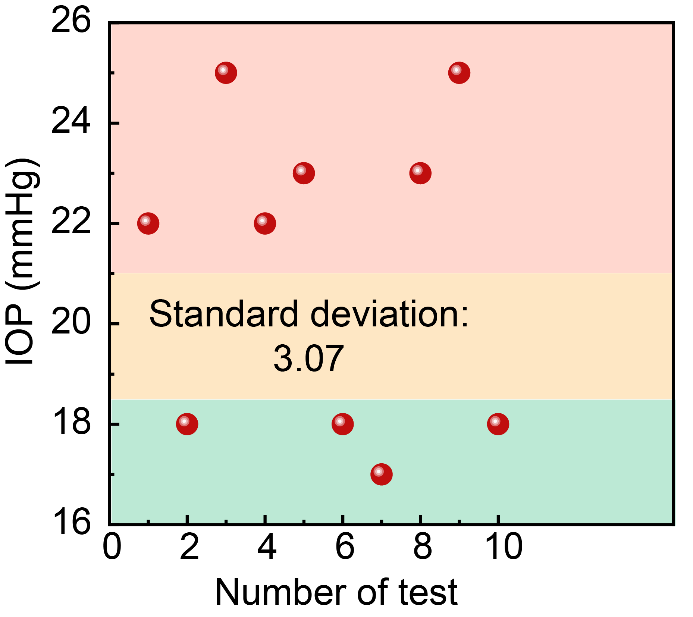


**Figure S35.** The results of ten consecutive measurements from a volunteer measured by using a commercial portable IOP tonometer (ICare IC100).

**Table S1.** Parameters of the 1DCNN model for the sensory system.

| No | Layer tape | No. of filters | Kernel/pool size | Stride | Input size | Output size | Padding |
| --- | --- | --- | --- | --- | --- | --- | --- |
| 1 | Convolution 1 | 32 | 4 | 1 | (None, 300, 10) | (None, 300, 32) | Same |
| 2 | Max-pooling 1 |  | 4 | 2 | (None, 300, 32) | (None, 150, 32) | Same |
| 3 | Convolution 2 | 64 | 2 | 1 | (None, 150, 32) | (None, 149, 64) | Valid |
| 4 | Max-pooling 2 |  | 4 | 2 | (None, 149, 64) | (None, 73, 64) | Valid |
| 5 | Convolution 3 | 128 | 2 | 1 | (None, 73, 64) | (None, 72, 128) | Valid |
| 6 | Max-pooling 3 |  | 4 | 2 | (None, 72, 128) | (None, 35, 128) | Valid |
| 7 | Flatten |  |  |  | (None, 35, 128) | (None, 4480) |  |
| 8 | Dense |  |  |  | (None, 4480) | (None, 20) |  |

**Table S2.** Parameters of our sensory system and their comparison with existing work.

| Ref | Sensor number | Model | Classification number | Epoch | Accuracy | Function |
| --- | --- | --- | --- | --- | --- | --- |
| 27 | 76 | CNN | 20 | 15 | 96.9% | Rescue missions |
| 17 | 15 | CNN | 20 | 50 | 86.7% | Sign language recognition |
| 18 | 15 | CNN | 28 | 50 | 97.14% | Object recognition |
| 19 | 12 | CNN | 10 | 50 | 96% | Floor monitoring system |
| 20 | 5 | CNN | 5 | 200 | 100% | Respiratory monitoring |
| 21 | 16 | CNN | 7 | 200 | 96.88% | Object recognition |
| 25 | 8 | LDA | 26 | 500 | 92.6% | Gesture recognition |
| 26 | 1 | RNN | 20 | 500 | 94.5% | Decoding lip language |
| 24 | 25 | SVM | 20 | 1000 | 98.6% | Object recognition |
| Our Work | 2 | CNN | 20 | 10 | 99.25% | Hardness recognition |

CNN: Convolutional Neural Network

LDA: Linear Discriminant Analysis

RNN: Recurrent Neural Network

SVM: Support Vector Machine

**Table S3.** IOP results of 25 volunteers measured using the jet measurement-based IOP tonometer (Non-Contact Tonometer NCT-200).

| Volunteer | Gender | Age | Left-IOP (mmHg) | Right-IOP (mmHg) |
| --- | --- | --- | --- | --- |
| 1 | Male | 20 | 17.0 | 18.0 |
| 2 | Male | 22 | 17.0 | 14.7 |
| 3 | Female | 22 | 18.0 | 15.3 |
| 4 | Male | 23 | 18.0 | 14.7 |
| 5 | Male | 23 | 19.3 | 18.7 |
| 6 | Male | 26 | 15.0 | 23.7 |
| 7 | Male | 28 | 16.0 | 17.0 |
| 8 | Male | 29 | 15.5 | 20.3 |
| 9 | Male | 30 | 18.7 | 15.0 |
| 10 | Female | 32 | 12.3 | 14.3 |
| 11 | Female | 36 | 15.0 | 18.0 |
| 12 | Male | 37 | 12.0 | 14.0 |
| 13 | Female | 45 | 13.3 | 14.7 |
| 14 | Male | 48 | 17.0 | 29.0 |
| 15 | Female | 50 | 15.3 | 15.3 |
| 16 | Male | 51 | 17.0 | 22.0 |
| 17 | Male | 52 | 19.0 | 16.0 |
| 18 | Female | 55 | 17.0 | 16.0 |
| 19 | Male | 57 | 17.0 | 18.5 |
| 20 | Male | 58 | 12.3 | 14.7 |
| 21 | Female | 59 | 10.0 | 10.0 |
| 22 | Female | 59 | 21.0 | 15.0 |
| 23 | Male | 60 | 19.3 | 15.0 |
| 24 | Male | 60 | 29.0 | 25.0 |
| 25 | Male | 60 | 15.0 | 15.0 |

**Table S4.** The parameters of the 1DCNN model for IOP monitoring.

| No | Layer tape | No. of filters | Kernel/pool size | Stride | Input size | Output size | Padding |
| --- | --- | --- | --- | --- | --- | --- | --- |
| 1 | Convolution 1 | 32 | 4 | 1 | (None, 300, 10) | (None, 300, 32) | Same |
| 2 | Avg-Pooling 1 |  | 4 | 2 | (None, 300, 32) | (None, 150, 32) | Same |
| 3 | Convolution 2 | 64 | 4 | 1 | (None, 150, 32) | (None, 150, 64) | Same |
| 4 | Avg-Pooling 2 |  | 4 | 2 | (None, 150, 64) | (None, 75, 64) | Same |
| 5 | Convolution 3 | 128 | 4 | 1 | (None, 75, 64) | (None, 75, 128) | Same |
| 6 | Avg-Pooling 3 |  | 4 | 2 | (None, 75, 128) | (None, 38, 128) | Same |
| 7 | Flatten |  |  |  | (None, 38, 128) | (None, 4864) |  |
| 8 | Dense |  |  |  | (None, 4864) | (None, 3) |  |

**Supplementary References:**

1. Meththananda I M, Parker S, Patel M P, *et al*. The relationship between Shore hardness of elastomeric dental materials and Young's modulus. *Dent Mater*, 2009; **25**: 956-959.
